# Effects of lysosomal biotherapeutic recombinant protein expression on cell stress and protease and general host cell protein release in Chinese hamster ovary cells

**DOI:** 10.1002/btpr.2455

**Published:** 2017-03-29

**Authors:** Damiano Migani, C. Mark Smales, Daniel G. Bracewell

**Affiliations:** ^1^ The Advanced Centre of Biochemical Engineering, Dept. of Biochemical EngineeringUniversity College LondonBernard Katz BuildingLondonWC1E 6BTUnited Kingdom; ^2^ Industrial Biotechnology Centre and School of BiosciencesUniversity of KentCanterburyKentCT2 7NJUnited Kingdom

**Keywords:** proteomics, host cell protein, proteases, acid alpha glucosidase, Chinese hamster ovary cells, biotherapeutic recombinant protein overexpression, downstream purification

## Abstract

Recombinant human Acid Alpha Glucosidase (GAA) is the therapeutic enzyme used for the treatment of Pompe disease, a rare genetic disorder characterized by GAA deficiency in the cell lysosomes (Raben et al., Curr Mol Med. 2002; 2:145–166). The manufacturing process for GAA can be challenging, in part due to protease degradation. The overall goal of this study was to understand the effects of GAA overexpression on cell lysosomal phenotype and host cell protein (HCP) release, and any resultant consequences for protease levels and ease of manufacture. To do this we first generated a human recombinant GAA producing stable CHO cell line and designed the capture chromatographic step anion exchange (IEX). We then collected images of cell lysosomes via transmission electron microscopy (TEM) and compared the resulting data with that from a null CHO cell line. TEM imaging revealed 72% of all lysosomes in the GAA cell line were engorged indicating extensive cell stress; by comparison only 8% of lysosomes in the null CHO had a similar phenotype. Furthermore, comparison of the HCP profile among cell lines (GAA, mAb, and Null) capture eluates, showed that while most HCPs released were common across them, some were unique to the GAA producer, implying that cell stress caused by overexpression of GAA has a molecule specific effect on HCP release. Protease analysis via zymograms showed an overall reduction in proteolytic activity after the capture step but also revealed the presence of co‐eluting proteases at approximately 80 KDa, which MS analysis putatively identified as dipeptidyl peptidase 3 and prolyl endopeptidase. © 2017 American Institute of Chemical Engineers Biotechnol. Prog., 33:666–676, 2017

## Introduction

Host cell protein (HCP) release during the production of therapeutic proteins is a critical quality attribute that must be monitored and reduced to acceptable levels during the production of a biotherapeutic protein.^1^ Being able to control HCP release during harvest, and their removal via downstream processing, is thus an important component of the manufacturing of such molecules and must be reported to the regulatory authorities. Typical purity targets for a monoclonal antibody (mAb) are <100 ppm HCP, <10 ng/dose DNA, and <5% product aggregates.^2^ Some HCPs are known to be co‐purified with the recombinant protein target, while others, specifically proteases, are reported to lead to product degradation.^3^ As a wide range of recombinant products are negatively affected by the presence of proteases such as reported for mAbs,^4^ fusion proteins,^5^ coagulation factors^6^ and based on personal communication with BioMarin who have manufacturing experience of GAA therapeutic enzyme products, we suspect GAA in certain conditions to also be similarly potentially affected.

Expression of therapeutic proteins from mammalian cell platforms presents multiple challenges associated with removal of both product related and host cell derived (e.g., proteins, DNA, RNA, lipids) impurities. It is believed that stress resulting from protein overexpression causes morphological changes to cell structure and an increase in HCP released, which can impact on downstream processing. As widely described in the literature, HCPs in final drug formulation can potentially have an impact on drug toxicity and patient safety.^7^

In addition to target molecule overexpression, another factor that can have an impact on cell stress is the high local energy dissipation rate associated with bubble bursting during culture, particularly in stirred bioreactors. A recent paper^8^ describes how cell death and lysis cause HCP release in the broth throughout the duration of the culture and are critical process parameters difficult to track as cell counters cannot account for lysed cells. The cell viability, cell size and choice of harvest day all therefore contribute to total HCP released due to shear during primary recovery.^9^

Historically, immunoassay‐based techniques such as ELISA have been used as high throughput methods to quantify HCP amounts during process development, manufacturing and in final product formulations. This approach has been important in the development of HCP assays and process development and product characterization; however, there are some inherent limitations with the use of ELISA. For example, the polyclonal antibodies used for detection are raised against the spectrum of HCPs found in the host cell (or in some cases mixed pools of cells), results are thus generally cumulative of all the HCPs present in a sample and cannot provide identification of single critical HCPs of interest, and only those HCPs which are immunogenic can be detected. These issues all potentially have impact as to whether all HCPs are being measured and with sufficient sensitivity.^1^, ^10^ The need to simultaneously quantify and identify HCPs in samples with greater sensitivity and precision, has resulted in the advancement of orthogonal analytical techniques based on LC‐MS/MS. High throughput monitoring of HCPs via LC‐MS/MS is becoming ever more common.^11^, ^12^

**Figure 1:**
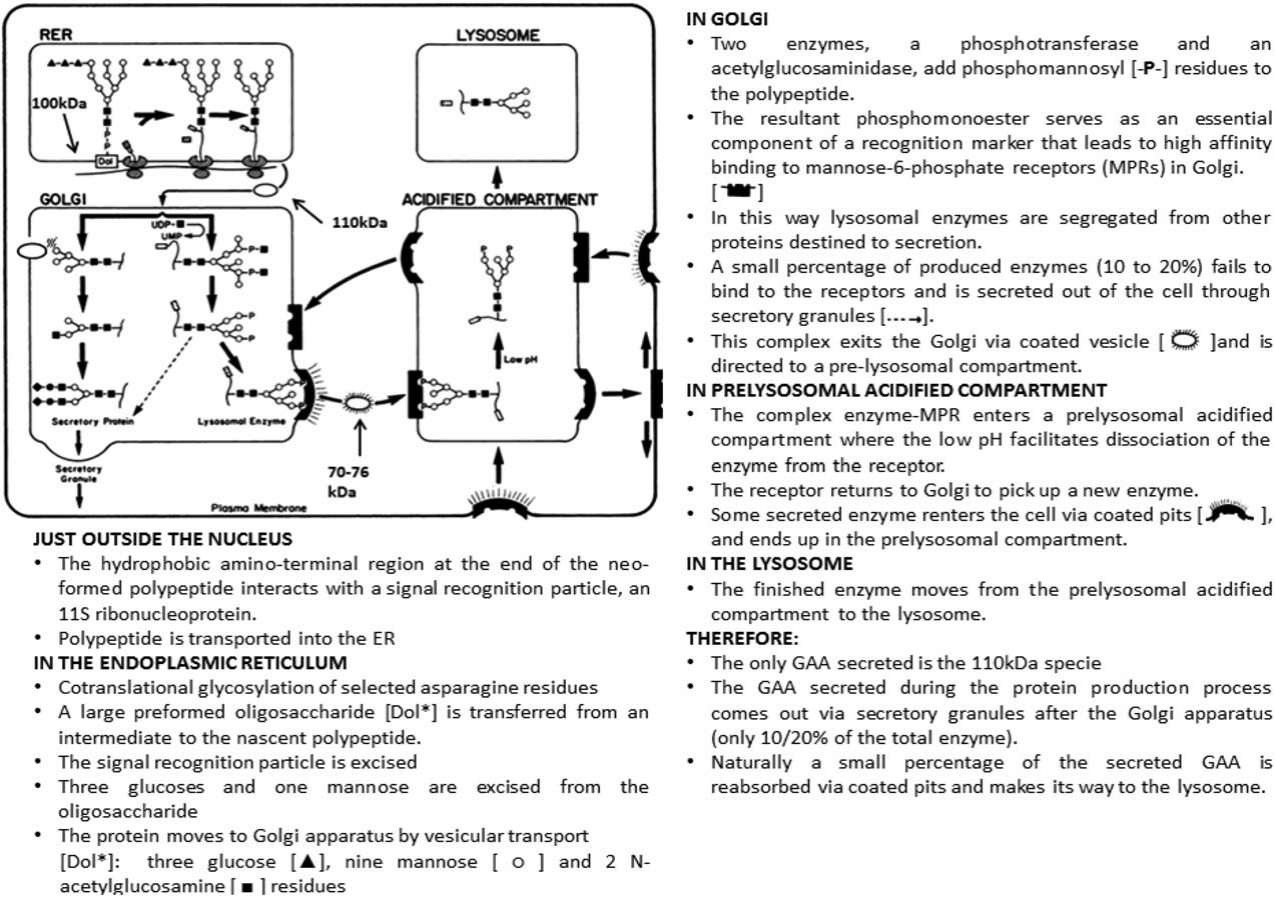
GAA Pathway with permission from The FASEB journal.^17^ Text adapted from Refs. 18, 19, and 20, with permission.

Here, we have specifically investigated the production of the lysosomal enzyme GAA in CHO cells and the impact on the HCP profile. To do this, rather than describing the full bioprocess involved in GAA purification, we have focused on HCPs present after the capture chromatographic step (IEX) to compare among three mammalian cell lines: a GAA, a mAb, and a null producer. The mAb producer was used as an example of another cell line overexpressing a recombinant protein to provide an alternate control to that of a null cell line. As seen in Swanson et al.^13^ IEX is an incredibly useful tool in clarifying complex mixtures of HCP, although to achieve an acceptable level of purification usually additional orthogonal techniques and depth filtration^14^ are used. In the case of GAA, the cysteine proteases Cathepsin B and Z are believed to be responsible for target molecule (GAA) degradation,^15^, ^16^ and it is therefore important to track their expression, release and removal throughout the bioprocess. According to the work reported by Kornfeld^17^ (Figure 1) as GAA enters the ER as a newly formed polypeptide, it undergoes post‐translational modifications in the ER and Golgi before approximately 10‐20% of enzyme is secreted outside the cell while the rest ends up in lysosome where it resides. It is hypothesized that while all mammalian cells produce a basal amount of GAA, the amount in recombinant overexpressing CHO cells will be much higher and this will have an effect on the phenotype of the lysosome. In this work, we use high resolution label free LC‐MS/MS to compare HCPs of three different CHO cell lines: a GAA producer, a mAb producer and a null. We also assess cell stress deriving from enzyme overexpression by comparing transmission electron microscopy (TEM) generated images of lysosomes, the organelles that store GAA.

## Materials and Methods

### Cell line generation

The site‐specific gene integration expression system Flp‐In/FRT (cat# V601020 ThermoFisher) was used to generate stable CHO clonal lines carrying the human GAA expression gene and HygromycinB resistance for selection. The GAA‐XL6 sequence (cat# SC125512 Origene) was cloned into the pcDNA/FRT plasmid using standard cloning techniques and the appropriate restriction enzymes (Not_1_ and Ase_1_), TSAP and ligase enzymes. OneShot TOP10 chemically competent *E. coli* cells (cat# V601020, ThermoFisher) were used for transformation and amplification of the genetic material following the manufacturer's protocol. Single colonies were picked from Petri dish and amplified overnight under vigorous shaking (250 rpm, 37°C) in LB ampicillin media. DNA was purified with the commercially available Qiagen Miniprep Kit (Qiagen Cat No.ID: 27104).

To ensure size and direction of insertion of the GAA gene were correct, a series of agarose gels were run of restriction digests and the plasmid was sequenced using custom primers (data not shown) that confirmed the correct sequence was present in the required orientation and in frame. The resulting plasmid DNA construct was used to transiently transfect the CHO Flp‐In cell line (Thermo Fisher) using FreeStyle™ MAX CHO Expression System (cat# K900020, Thermo Fisher). Following confirmation of the presence of GAA in transiently transfected culture supernatant via western‐blot analysis, stable cell line generation was performed. A pcDNA/FRT‐GAA construct was used to transfect the CHO Flp‐In (cat# R75807, Thermo Fisher Scientific) commercially available cell line that had been previously adapted to grow in chemical defined CD‐CHO media (ThermoFisher) + 8 mM glutamine, using PEI (Polyethylenimine linear, cat#9002‐98‐6 Sigma Aldrich) as a transfection agent and the pOG44 Flp‐Recombinase Expression Vector (cat# V600520 Thermo Fisher Scientific) in a (1:9):3 ratio (3 µg of plasmid DNA added to 27 µg of pOG44, incubated 5 min at RT, followed by 90 µg of linear PEI). Colonies that emerged under 250 µg/mL hygromycin B selection pressure were subject to limiting dilution cloning (LDC). A total of 360 wells were plated of which only 6 ultimately grew, and eventually only 3 were viable. The three final cell lines were assessed for GAA titer and growth performance. Cell counting and viability was monitored using a Beckman Coulter ViCell while GAA titer was measured via Okumiya GAA diagnostic assay method,^21^ using a GAA reference standard for activity and concentration comparison.

### Lysosomal imaging using TEM

Lysosome images were collected using a Joel 1010 TEM with Orius Gatan camera system. Small aliquots (150 µL of 10^6^ viable cell/mL) of null CHO and GAA CHO cells were taken aseptically from culture shake flasks, immediately centrifuged (2000 g, 5 min) and the supernatant removed. A solution of 2% paraformaldehyde, 1.5% glutaraldehyde in 0.1 M cacodylate buffer pH 7.3 was added to the pellet and incubated at 4°C. After a series of washes (0.1 M cacodylate 5 min, H_2_O 5 min, 0.5% uranyl acetate 20 min, 0.1 M cacodylate 5 min, H_2_O 5 min) pellets were dehydrated via increasing concentration ethanol washes and eventually fixed onto epoxy resin (12 g agar, 8 g dodecenylsuccinic anhydride, 5 g methyl nadic anhydride, 0.65 mL N‐benzyldimethylamine, Sigma Aldrich) and hardened for 24 h at 60°C. Ultrathin slices of 70 nm were prepared using a diamond knife on a Reichetr ultracut E microtome and collected on a 200 mesh copper grids. Finally, sections were stained with uranyl acetate and lead citrate. In total 33 images of single cells per sample were taken. Each image was analyzed for lysosome number and condition. Lysosomes observed were classified in three categories: Full (F) if the lysosome seemed to be completely full with matter, Half Full (HF) if the internal structure was partially filled or Empty (E) if the structure appeared to be clear of any matter visually. The numbers were collated in Excel and the following analysis conducted: (1) Total number of lysosomes per cell; (2) Number of E, HF, F counted per cell; (3) Standard Deviation over population; (4) Standard Error over population.

### Cell culture

A cell bank vial (1 mL 10^7^ cells) was thawed from liquid nitrogen in 37°C water bath and quickly resuspended in 10 mL of fresh pre‐warmed CD‐CHO media. DMSO was removed by 2000 g, 5 min centrifugation and resuspension in 35 mL of pre‐warmed media containing 250 µg/mL Hygromycin B in a 125 mL cell flask. Three days of revival, cells were passaged to 0.3 10^6^ cell/mL into 150 mL of fresh media in half litre bottom‐baffled shake flasks. Flasks were incubated at 37°C 5% CO_2_ on an orbital shaker (125 rpm), until they reached approximately 10 million cells/mL in 8 days. Viability was followed so that this did not drop below 95%. On the day of harvest, cell broth from the flask was transferred to 3 × 50 mL conical tubes and centrifuged at 2000 g for 10 min to pellet the cells. The supernatant was filtered through a 0.22 um stericup. Harvest Cell Culture Fluid (HCCF) was then concentrated 4‐fold using multiple 10,000 MWCO (cat# 88527, ThermoFisher Scientific) centrifugal concentrators (to approximately 30 mL).

### Western blot for product detection

Proteins in clarified HCCF samples were fractionated by SDS‐PAGE and transferred to a PVFD membrane using an XCell II Blot module according to the manufacturer's protocols (Invitrogen). After incubation with 5% non‐fat milk in PBST (Phosphate Buffered Saline Tween‐20) for 60 min, the membrane was washed once with PBST and incubated with rabbit raised antibodies against GAA (1:1000) (cat.# ab137068 Abcam) at 4°C for 12 h. Membrane was then washed three times for 10 min in PBST and incubated with a 1:1000 dilution of horseradish peroxidase‐conjugated anti‐rabbit antibody for 1 h at RT. Membrane was washed with PBST three times and a diaminobenzidine detection kit (Sigma‐Aldrich) was used to highlight areas of GAA presence.

### GAA purification first capture step: anion exchange chromatography

A GE AKTA Avant 25 (GE Healthcare Life Sciences) system was used to set up the anion exchange chromatography capture step. The column used was a 1 mL GE CaptoQ HiTrap and the buffers as follows; equilibration buffer 50 mM sodium acetate pH 6.0 (Buffer A), elution buffer 1 M sodium chloride in 50 mM sodium acetate pH 6.0 (Buffer B) at a flowrate of 1 mL/min. Concentrated HCCF was diluted 1:4 into equilibration buffer to adjust conductivity and favor binding of target material to resin. A 30 mL volume was then loaded onto the column followed by a 14 CV wash (100% buffer A), 40 CV linear gradient elution (0‐35% buffer B), 5 CV strip (100% Buffer B) and regeneration (10 CV 0.5 M NaOH, 5 CV Buffer A, 10 CV 20% EtOH). 2 mL fractions of column wash and eluate were collected and absorbance at 280 nm monitored throughout the run. The eluate was immediately frozen at −20°C until further analysis.

### The use of Zymograms to follow and identify protease activity

Novex™ 12% Zymogram Casein Protein Gels (cat# EC6405BOX Thermo Fisher Scientific) were used to visualize protease presence pre‐ and post‐ capture step in null and GAA CHO cells. Samples were first denatured in SDS buffer and separated based on size similar to a standard SDS‐PAGE gel, then they were renatured by incubating the gel in non‐ionic detergents (renaturing buffer, Invitrogen) to re‐establish enzymatic activity. The gel was then incubated overnight at RT in developing buffer (Invitrogen) which adds divalent metal cations required for enzymatic activity. The following day the gel was stained with Comassie blue and de‐stained in 40% MeOH, 10% acetone. Regions of protease activity appear as clear bands against a dark blue background where the protease has digested the substrate.

### HCP quantification via AlphaLISA

HCP content in HCCF and post capture chromatography eluates was measured via Perkin Elmer AlphaLISA using a commercially available CHO HCP (broad reactivity) AlphaLISA Detection Kit (cat# AL301C) read on an Envision reader (Perkin Elmer) at 680 nm excitation/615 nm emission following the manufacturers' protocol.

### HCP identification and quantification using high resolution nanoLC‐MS/MS

Eluate fractions post anion exchange from the three cell lines (GAA Cell line1, null and mAb producing) were screened via nanoLC tandem MS to determine HCP profile and presence of product. LC‐MS/MS analysis was undertaken by first digesting samples with trypsin as described below, separating digested peptides on UPLC and lastly injecting them into the mass spectrometer. For digestion, post‐AIEX column eluates fractions were concentrated via 10000 MWCO concentrators (Millipore) by a factor of 4. 50 µL of sample were mixed with 8 M urea in 100 mM NH4HCO_3_ + 1 µL 450 mM DTT. This was allowed to incubate at RT for 1 h and followed by 15 min incubation at RT with 10 µL 100 mM iodoacetamide. Final protein concentration was determined with a NanoDrop instrument at 280 nm. The urea concentration was then diluted with 64 µL of HPLC grade water. Digestion with trypsin was performed for 1 h at 37°C by adding to each sample 4 µg of sequencing grade Trypsin (Promega V5111). A 2 mg/mL BSA (Thermo Fisher) stock solution was used as a quantitation standard. 50 fmol of BSA was prepared by serial dilution and spiked into each sample prior to separation to allow for label free quantitation.

For peptide separation, 10 μl of sample was injected in triplicate onto a HSS T3 Acquity column (Waters) 75 μm internal diameter × 15 cm (1.8 μm, 100 A) for on‐line reverse phase UPLC (Acquity M‐Class, Waters). Elution was performed with a linear gradient from 3 to 40% B over 40 min; (buffer A: 0.1% formic acid in water. Buffer B: 0.1% formic acid in acetonitrile) at a flow rate of 300 nl/min. The M‐class mass spectrometer was coupled via a nanospray source to a Synapt G2‐Si (Waters) and data were collected in HDMSe mode. Data were analyzed using Progenesis QIP and searched against SwissProt using MSe Search with a false detection rate of 4%. Progenesis QIP software provided resulting data in the form of normalized average abundance (molar equivalent) of each species matched in the SwissProt database in triplicate readings. The average of each triplicate was calculated eliminating eventual outliers. Average abundances were converted to mass by multiplying them by the respective molecular weights. Score and probability values were used to avoid identification of false positives. In particular Anova (*P*) probability factor threshold was set at 0.1 max, which means that at *P* of 0.1, there is a 10% chance of a false positive being identified.

Proteins identified were checked individually against the UniProt database using accession IDs to determine biological functions (proteolytic activity). Isoelectric point was calculated using ExPASy pI calculator online tool (ExPASy. Compute pI/Mw tool). Hydropathicity index (GRAVY) was also calculated based on amino acidic sequence. Relative percentage of each protein out of the total were also calculated and used for the resulting bubble graph diagram.

## Results and Discussion

### Clonal CHO cell line generation

Generation of stable recombinant protein expressing mammalian cell lines was traditionally a long and tedious task, mainly due to the bottleneck operation of clone selection, however with the advent of high throughout and cell sorting technologies this has become a much more stream lined procedure such that stably expressing clones can typically be established in 3‐4 months.^22^ Transfection of a plasmid into a genome is a rare random event and a very low number of clones, usually 1 in 10,000^23^ will present the desirable features of high titre and good quality product. Due to this “location effect,”^24^ the odds that a plasmid integrates in highly transcribed section of the host genome (away from chromatin or other less transcribed areas) are very low. It thus becomes necessary to screen thousands of clones to select high producers.

In this work, we opted to use an alternative technique based on site‐directed recombination using Flp‐In/FRT technology, which facilitates integration of the gene of interest into a specific highly transcribed FRT site in the host genome and allows all clones generated to produce considerable and comparable levels of recombinant product.^25^ Using this technology, and the process outlined in the material and methods section, three GAA producing cell lines were established using LDC.

**Figure 2:**
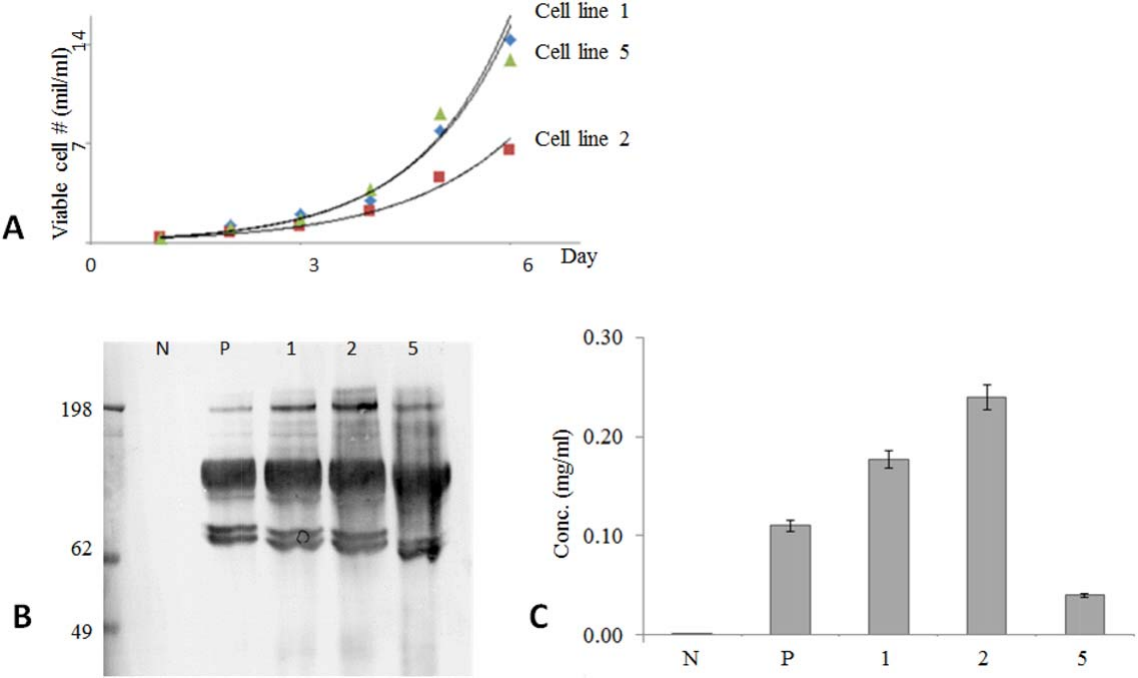
CHO GAA producing and control cell line comparison. (A): cell growth performance; (B): product production via western blot; (C): product production quantification via GAA diagnostic assay.^21^ Although cell line 2 was the highest producer, cell line 1 was the fastest grower. Cell line 1 was chosen due of good balance between growth and production performance. Data show that null (N) cell line produces non detectable product as expected, and pool sample (*P*) secretes average level of GAA. Pool sample is the sample used for cell line selection (all cell lines mixed). Medium used ThermoFisher scientific CD‐CHO with 250 µg/mL HygB, viability >93% at day 6 (null grown in same media without HygB).

The three cell lines (GAA, mAb, and Null) were compared in terms of growth performance (Figure 2A) and this showed that the highest GAA producing cell line (#2) had the slowest growth over a 6‐day period, which may reflect the impact of GAA expression on this cell line. Cell line 1 and 5 grew very similarly and reached viable cell numbers of 14 million per mL over the same 6 day period. Western blot analysis of the HCCF (Figure 2B) confirmed the presence of the 106 KDa GAA isoform in all three cell lines as well as in the pool sample (heterogeneous cell population that emerged from stable transfection selection pressure before applying LDC) and undetectable amounts of GAA in an untransfected negative control (null). We also observed in the same samples the presence of smaller GAA isoforms, which could be the 70 and 76 KDa species described in the literature.^18^ A GAA diagnostic assay that uses glycogen and 4‐methylumbelliferyl‐α‐D‐glucopyranoside as substrates for measuring GAA activity and incorporates acarbose to eliminate the interference of unrelated α‐glucosidases^21^ was used for quantification of GAA in HCCF samples (Figure 2C). This assay showed a range of expression from approximately 0.05 to 0.25 mg/mL of GAA. These data are in accordance with the western blot data. After having assessed these factors, cell line 1 was selected for further studies as it produced 0.18 g/l of GAA in harvest and was capable of reaching 14 million cell/mL in 6 days.

### Investigating the lysosomal phenotype with TEM in GAA producing cells

**Figure 3:**
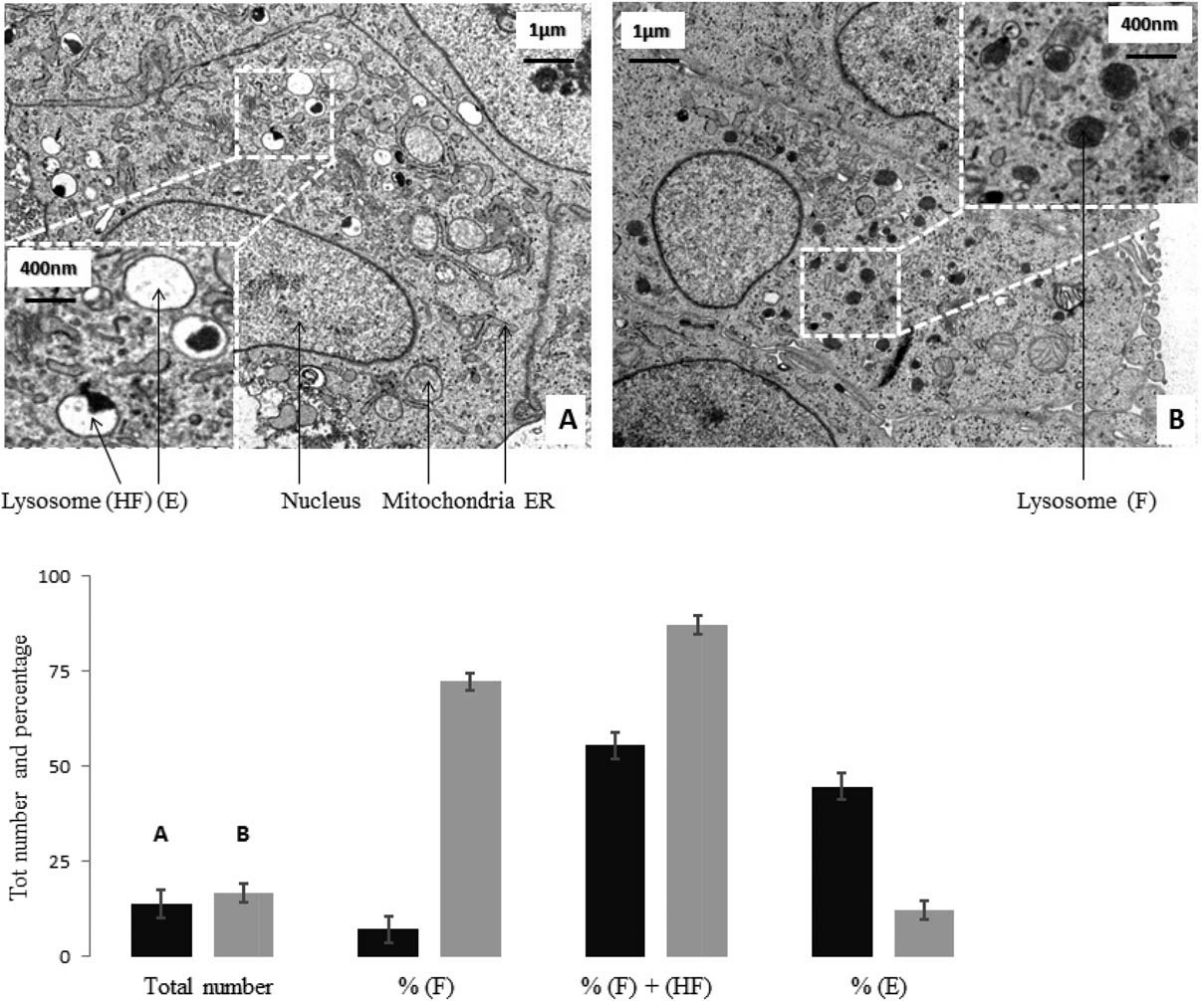
Transmission Electron Microscope images of null (A) and GAA (B) cells. Cells were sampled at the same time from actively growing cultures, fixed and ultra‐thin slices were prepared for TEM imaging (magnification 3000X). 33 images of single cells were taken per each sample. Lysosomes were counted and categorized based on content in Full (F), Half‐Full (HF) and Empty (E). Data were collated and analyzed in excel (black – null; gray – GAA). Images chosen are representative.

After establishing that the GAA cell lines were expressing the desired product, we next investigated any potential influence of this on the lysosomes of the recombinant cells compared to the host. As explained in Figure 3, all mammalian cells produce a basal amount of GAA, 10 to 20% of which is secreted outside of the cell. The rest is transported to pre‐acidified compartments via mannose 6 phosphate binding and is thus transported into the cells lysosomes. We wanted to determine if GAA overexpression affected lysosomal morphology by comparing images of lysosomes from the recombinant GAA expressing cell line and the originating null CHO cells. The resulting data are presented in Figure 3 and shows a clear difference in lysosomal phenotype between null and GAA cells. While lysosomes are present in similar numbers (about 18 per cell) and size (400 nm) in both cell types, they are generally full and dark in recombinant GAA and empty or mostly empty in null cells. Specifically, over 72% of lysosomes are completely full (F) in the GAA cell line while only 8% present the same phenotype in the null line. This trend was also observed when comparing lysosomes containing some (%HF – half full) to no (%E ‐ empty) dark matter: only 17% of all lysosomes in the GAA expressing cell line present this phenotype vs. 55% in null. These data provide evidence that overexpression of GAA leads to lysosomes becoming full and engorged. Potentially this could contribute to the overall cellular stress levels experienced by the cell.

### Purification of recombinant GAA

To investigate how cell stress and HCP content in the cell culture supernatant are linked to target protein overexpression, we first designed an anion exchange step to capture GAA from the clarified harvest feed. Previous attempts at alpha‐amylase purification involved anion‐exchange chromatography using mono‐Q columns^26^ and affinity chromatography using Concanavalin A Sepharose 4B for GAA.^27^ For this experiment, we decided to use anion exchanger Capto‐Q.

**Figure 4:**
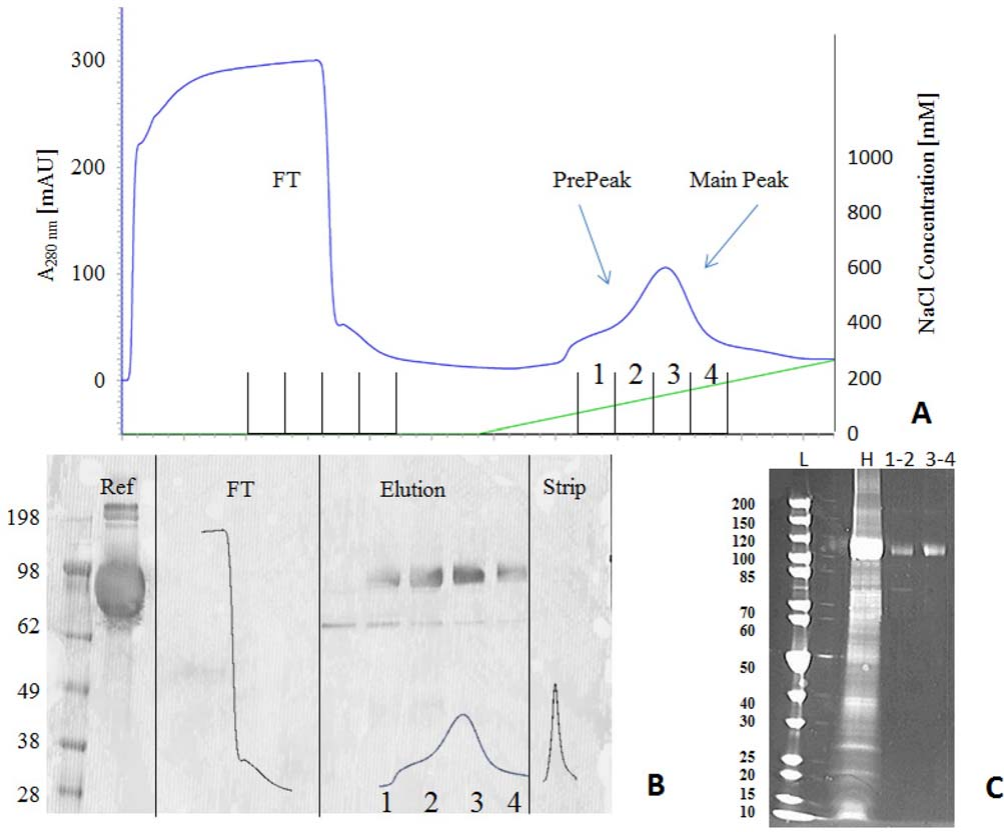
Elution peak in AIEX chromatogram (A) is divided in four fractions and analyzed. Western blot analysis (B) shows absence of GAA in flowthrough and strip, and its presence in the four elution fractions, with the main amount eluting in fraction 2 and 3 (prestained molecular weight standard cat# LC5925 Thermo Fisher). For SDS‐PAGE SYPRO ruby (C) fractions were combined (1–2 and 3–4). These data show presence of GAA at around 110KDa in both fractions together with lower molecular species in minor quantity (ladder molecular weight standard cat# 26614 Thermo Fisher). Clarified Harvest (H) was also run along for comparison.

After growing the cell line in 100 mL CD‐CHO, 8 mM glutamine, HygromycinB containing media in cell flaks up to 15 × 10^6^ cells, the batch culture was harvested and partly clarified via centrifugation. The GAA in the HCCF was then partly purified via anion exchange capture step on an AKTA Avant using CaptoQ resin. As can be seen in Figure 4A, GAA eluted at a salt concentration range between 100 and 200 nM. Fractions were collected during flowthrough, elution and column strip and GAA presence was assessed via western blot analysis. In Figure 4B, GAA was present in varying concentrations only in the four elution fractions and no GAA was observed in flowthrough and strip fractions. The GAA bands were slightly below the expected molecular weight for GAA (106 KDa as observed previously and in MS data below), however so was the reference standard. This can be attributed to the molecular weight standard being compromised or inaccurate. The same fractions analyzed via SDS‐PAGE (Figure 4C) further show the degree of purification achieved by the ion exchange step. By comparing the HCCF and IEX elution fractions (combined as illustrated), it is possible to visually observe the degree of HCP removal by IEX. Target protein recovery was measured at 89% via the GAA diagnostic assay.

**Figure 5:**
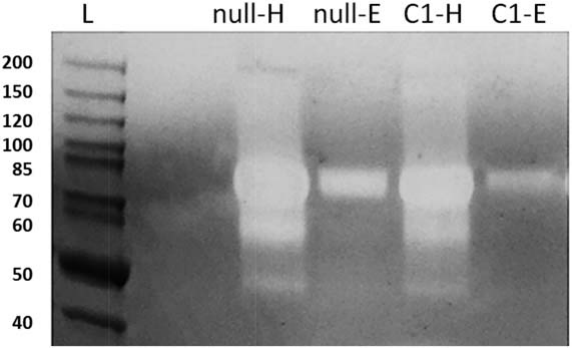
Comassie blue stained casein Zymogram. From left: null HCCF (null‐H), null AIEX Eluate (null‐E), Cell line1 HCCF (C1‐H), Cell line 1 AIEX Eluate (C1‐E). Protein samples are first separated according to molecular weight via gel electrophoresis, then renatured and incubated overnight in the gel containing casein substrate and finally stained and de‐stained. Areas that show proteolytic activity appear as white bands over dark background. Data show that AIEX is able to clear out the majority of proteases from HCCF to Eluate sample except for some co‐eluting ones at around 80 KDa. These were later putatively identified via MS as Dipeptidyl peptidase 3 and Prolyl endopeptidase. Furthermore, amount of protease in GAA cell line 1 eluate seems to be less than amount of proteases present in null eluate. Note: GAA, not being a protease, is not visible in zymography.

### Protease characterization in material from the GAA and null cell line using zymography analysis

Protease content in HCCF and elution fractions was compared between the selected GAA cell line and its originating null cell line to see whether overexpression of the target protein influences total protease activity and content. Initially, a zymographic gel (Figure 5) gave a qualitative representation of eluate protease content as it showed how the null eluate contained more proteases than its GAA clonal counterpart around the 80 KDa size. According to MS identification data shown in the following results section (see also Supporting Information – “HCP full list,” for complete HCP identification), the only two proteases of ∼80 KDa size present in both null and GAA IEX eluates are dipeptidyl peptidase 3, a metalloproteinase, and prolyl endopeptidase, a serine peptidase. The implications of the presence of these two proteases coeluting with the product has not been evaluated. This result called for further investigation, specifically identification and quantification of those proteases present that might impact on the quality of the final GAA product.

### HCP characterization in a GAA expressing CHO cell line compared to the null host and a monoclonal antibody producing cell line as determined by high resolution LC‐MS/MS

**Figure 6:**
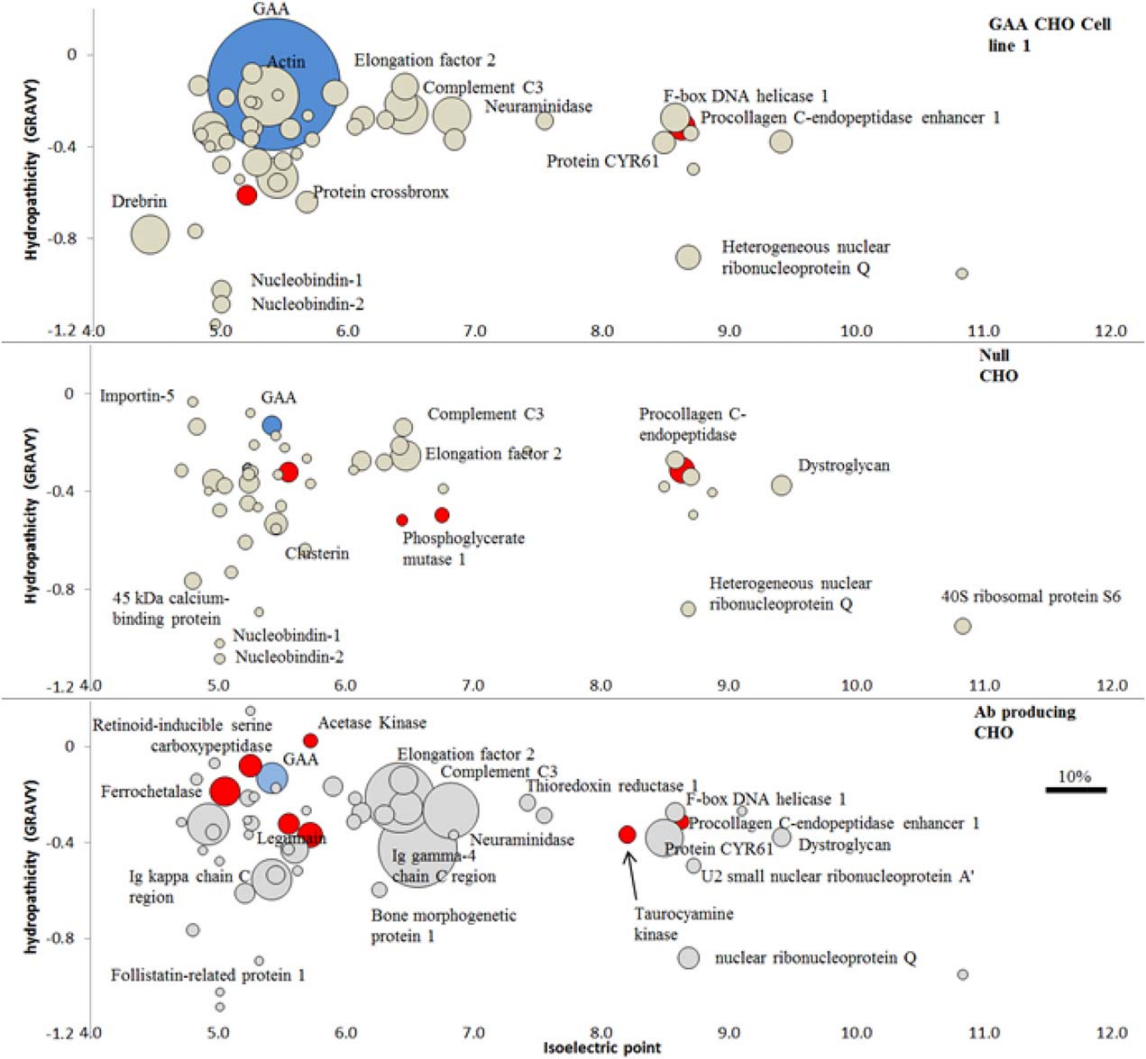
High resolution LC‐MS/MS. In blue target molecule GAA, in red HCPs with proteolytic activity, in grey all other HCPs. Data show identification and quantification of HCP found in capture step (IEX) eluate fraction across the three cell lines (GAA cell line1, null and mAb producing). Out of a total of 233 HCPs identified in the samples, 36 showed proteolytic activity. The first 50 more abundant species are reported per each cell line and ordered by calculated pI (ExPASy Compute pI/Mw tool, SIB Swiss Institute of Bioinformatics) on the *X* axis and by hydrophobicity index GRAVY (ExPASy ProtoParam tool, SIB Swiss Institute of Bioinformatics) on the *Y* axis. Bubble size represents percent of a given protein out of the total mass and was calculated by multiplying normalized abundance (averaged among triplicate LC injections) by the protein molecular weight. Post AIEX column eluate fractions were concentrated and digested with sequencing grade modified trypsin, 50 fmol of BSA standard was spiked in each digested sample before injection in LC to allow for label free quantitation. Separation via HSS T3 Acquity column (Waters) 75 μm internal diameter × 15 cm (1.8 μm, 100A) on‐line reverse phase UPLC (Acquity M‐Class, Waters). Elution via linear gradient from 3 to 40% B over 40 min; (buffer A: 0.1% formic acid in water. Buffer B: 0.1% formic acid in acetonitrile), flow rate 300 nl/min. Separated peptides were injected via nanospray source to a Synapt G2‐Si (Waters), data collected in HDMSe mode. Data analyzed using Progenesis QIP and searched against SwissProt using MSe Search and a false detection rate of 4%. Complete list of HCP identified in all samples is included in Supporting Information – “HCP full list.”

In this experiment, we compared HCP content found in capture step eluate fraction of three different CHO cell lines. The GAA producing cell line 1, the null and the mAb producing were expanded and purified under the same conditions and IEX eluate fractions were screened using high resolution nanoLC tandem mass spectrometry. The resulting data are presented in Figure 6 and Table 1. The data are shown in Figure 6 as a bubble graph generated by plotting the 50 most abundant species found in each cell line. These were ordered by calculated pI (ExPASy Compute pI/Mw tool, SIB Swiss Institute of Bioinformatics) on the *X* axis and by hydrophobicity index GRAVY (ExPASy ProtoParam tool, SIB Swiss Institute of Bioinformatics) on the *Y* axis. Bubble size (diameter) represents percent of a given protein out of the total mass and was calculated by multiplying normalized abundance (averaged among triplicate LC injections) by the protein molecular weight. The relative quantification calculation of each protein identified through this type of analysis is achieved via direct comparison with the BSA reference standard spiked in the samples before sample injection. In order for quantification to be absolute there should be a reference standard per identified ion or protein species, a clearly unfeasible solution. For this reason, we are confident of HCP ID being correct, even though the amounts of each protein are relative to the BSA control.

**Table 1:** Overlapping and Unique HCPs

**Name** (μg/ml)	**GAA**	**null**	**Ab**
Clusterin	3.5	7.6	0.2
Complement C3	3.4	13.5	0.6
Neuraminidase	2.8	0.1	1.6
Elongation factor 2	2.2	4.8	2.5
F‐box DNA helicase 1	1.7	4.9	0.2
EGF‐containing fibulin‐like extracellular matrix protein 1	1.7	7.0	0.1
Pigment epithelium‐derived factor	1.4	5.1	0.4
Procollagen C‐endopeptidase enhancer 1	1.4	9.4	0.1
Heterogeneous nuclear ribonucleoprotein Q	1.2	3.2	0.2
Dystroglycan	1.0	6.1	0.2
Protein CYR61	1.0	1.6	0.7
Follistatin‐related protein 1	0.8	2.9	0.2
Retinoid‐inducible serine carboxypeptidase	0.8	1.4	0.2
Lysosomal protective protein	0.8	5.8	0.2
Ιmportin‐5	0.8	4.3	0.1
Nucleobindin‐1	0.7	1.3	0.0
Dipeptidyl peptidase 3	0.7	2.6	0.1
Nucleobindin‐2	0.6	1.6	0.0
Cathepsin B	0.6	1.8	0.0
78 kDa glucose‐regulated protein	0.6	2.7	0.0
Nidogen‐1	0.5	5.7	0.0
cAMP‐dependent protein kinase catalytic subunit gamma	0.5	4.7	0.0
Legumain	0.4	1.4	0.3
45 kDa calcium‐binding protein	0.4	3.9	0.1
Actin‐17	0.3	1.5	0.1
U2 small nuclear ribonucleoprotein A'	0.3	1.2	0.1
Actin‐10	0.3	0.4	0.0
Spermidine/putrescine import ATP‐binding protein	0.3	1.0	0.0
Putative phospholipase B‐like 2	0.2	1.2	0.0
40S ribosomal protein S6	0.2	3.7	0.1
Actin, gamma	0.2	1.4	0.1
Collagen alpha‐l (VI) chain	0.2	0.9	0.0
Bone morphogenetic protein 1	0.2	0.9	0.1
Annexin A5	0.2	0.3	0.0
Rho GDP‐dissociation inhibitor 1	0.2	2.3	0.0
Receptor‐type tyrosine‐protein phosphatase S	0.2	1.5	0.0
Nuclear migration protein nudC	0.2	0.3	0.0
Heat shock cognate 71 kDa protein	0.1	3.9	0.0
30S ribosomal protein S6	0.1	0.2	0.0
Heat shock 70 kDa protein	0.1	0.9	0.0
Small ubiquitin‐related modifier 2	0.1	1.1	0.0
Chaperone protein dnaK2	0.1	2.4	0.0
Vitamin K‐dependent protein S	0.1	0.6	0.0
POTE ankyrin domain family member E	0.1	0.0	0.0
Transmembrane protein 132A	0.1	0.8	0.0
Matrix metalloproteinase‐19	0.1	1.3	0.0
Protein disulfide‐isomerase	0.1	1.0	0.0
Cathepsin Z	0.1	0.4	0.0
Actin	7.7	0.0	0.0
Drebrin	3.0	0.4	0.0
Probable dual‐specificity RNA methyltransferase RlmN	0.9	0.0	0.3
EGF‐containing fibulin‐like extracellular matrix protein 1	0.4	0.0	0.1

List of the 50 most abundant overlapping HCP common to all three cell lines (top) and four unique to GAA HCP (bottom). Values (calculated µg/mL) are ordered by abundance in GAA sample. HCP are considered “overlapping” when present in at least two out of three cell lines. Concentration (µg/mL) is calculated by multiplying normalized abundance value by molecular weight divided by sample volume (10 µL). Normalized abundance value based on BSA reference standard

As expected in cell line 1, GAA was the dominating species in the IEX eluate accounting for over 36% of the total by mass spectrometry. In the same GAA eluate sample, HCP quantified via AlphaLISA (PerkinElmer, MA) accounted for approximately 54% of total protein, in agreement with the MS result above (data not shown). By comparison, GAA accounted for approximately 2% by MS of the total species found in null and mAb producing cell lines, further proof that basal GAA levels are found across many mammalian cells lines and that the GAA cell line is expressing recombinant material. Actin, clusterin, drebrin, elongation factor 2, and lysosomal protective protein (carboxypeptidase) are some of the more common HCPs co‐purifying due to their pI being within 1 unit of the target molecule. HCPs with similar hydrophobicity to GAA but over 1 pI unit difference were found to be passenger molecules that co‐purified, some most likely by “product‐associated” interaction. This phenomenon of co‐purification with the product due to interaction with the product itself (piggy backing), has been described previously for antibodies.^28^, ^29^ In this category, we find: neuraminidase (pI 6.82), the protease involved in cleaving glycosidic linkages in neuraminic acids; procollagen C‐endopeptidase enhancer 1 (pI 8.63) involved in cartilage and bone formation; 40S ribosomal protein S6 (pI 10.83); the kinase cAMP‐dependent protein kinase catalytic subunit gamma (pI 8.7); and dystroglycan (pI 9.4) a complex involved in numerous processes such as cell survival and migration and membrane assembly (source Uniprot.org).

In the antibody producing cell line IEX eluate, some of the most abundant species found were antibody as expected, accounting for about 25% of the total by MS. In the pI range between 5 and 6, where direct adsorption to the IEX resin is anticipated, other proteins found include: legumain, a protease involved in lysosomal protein degradation; thioredoxin a protein responsible for various redox reactions through the reversible oxidation of its active center, and proteases lysosomal protective protein and ferrochelatase. Outside the typical pI range for direct interaction and therefore candidates for piggy‐backing through the process are: CYR61 (pI 8.49), which promotes cell proliferation angiogenesis and cell adhesion; neuroaminidase (pI 6.8), dystroglycan (pI 9.4) and 40S ribosomal protein S6 (pI 10.8).

In the null profile range of HCPs with pI between 4.5 and 6.5, we observed clusterin, lysosomal protective protein, Complement C3, thioredoxin (disulfide oxidoreductase), protocollagen C‐endopeptidase enhancer (peptidase), phosphoglycerate mutase (kinase), lysosomal protective protein (serine carboxypeptidase), legumain (lysosomal hydrolysis), and the common heat shock cognate protein 70 KDa.^30^ HCPs with pI > 6.5 were identified as dystroglycan (pI 9.4), 40S ribosomal protein S6 (pI 10.8), matrix metalloproteinase‐19 (pI 8.9). Interestingly most of the HCPs identified in the pI > 6.5 range in this sample, were common with those found in GAA and mAb producer, a sign that their elution was not caused by product associated interaction, as product in the null cell line is barely present. We believe that they might interact with another HCP that interacts with the resin.

Importantly, the cysteine protease cathepsin B and Z were ubiquitously present in moderate quantity in all three cell lines. More precisely cathepsin B was 0.60 μg/mL or 0.62% of total protein content in GAA producer, 1.85 μg/mL or 0.83% of total protein in null and 0.04 μg/mL or 0.20% of total protein in mAb producer. Cathepsin Z was 0.10 μg/mL or 0.10% of total protein in GAA producer, 0.41 μg/mL or 0.18% of total protein in null and 0.01 μg/mL or 0.05% of total protein in mAb producer. This suggests these are not co‐purifying with the products and are retained to some extent by the resin, or, that they also co‐purify with another HCP that does interact with the resin. Such proteases may present issues with the production of GAA.

With the exception of a small number of unique species found in excess in the GAA line and reported in Table 1, the HCP profile across the three cell lines was broadly similar and in agreement with other studies.^31^ A list of the 50 most abundant overlapping proteins common to all three cell lines is found in Table 1. This overlap in HCPs across many different cell lines has been studied extensively in two recent studies. In the first one,^9^ the authors compared HCPs in harvest supernatants of day 10, 12, and 14 cultures generated by a null cell line and a recombinant IgG4‐producing cell line and reported that while there were increases in HCPs, which they associated with viability decline later in culture, there were many similarities between the cell lines and days of harvest. In another study,^32^ the HCP profile of three different null cell lines was compared and it was reported that despite differences in CHO lineage, upstream process, and culture performance, the cell lines yielded similar HCP profiles. Furthermore, this study assessed that about 80% of the most abundant 1000 proteins identified were common to all three lines.

While the HCP profile in this study is largely similar across the three cell lines, some HCPs are unique to the GAA cell line. Table 1 (bottom) shows the relative distribution and calculated concentration of the four species that were found to be particularly abundant in the GAA line and almost absent in the other two. These are: (1) actin (cytoskeletal protein ubiquitously expressed in all eukaryotic cells) where a relatively low amount was found in null and mAbs lines, a much greater amount was found in GAA line material; (2) drebrin (related to actin polymerization) which was also present predominantly in the GAA cell line material; (3) Probable dual‐specificity RNA methyltransferase RlmN (involved in RNA formation); and (4) EGF‐containing fibulin‐like extracellular matrix protein 1 (involved in cell adhesion and migration). We suspect that this might be due to a combination of two factors: the large amount of GAA in cell line 1 sample could possibly be responsible for mass spectrometer detector sensitivity saturation, which could explain quantification discrepancies with the other two lines. This could also account for differences in the mAb cell line where a high concentration of mAb is present. Also, the competitive behavior of different species onto the anion exchange resin could have affected the amount of target bound coeluting species.

## Conclusions

In this work, we provide evidence that overexpression of a lysosomal therapeutic recombinant protein impacts on the lysosomal phenotype of a CHO cell line. We achieved this by comparing cell lysosomal phenotype and HCP profile of three CHO cell lines: a lysosomal biotherapeutic protein producer (GAA), a mAb producer and a null. The analytical approach based on lysosomal phenotype image analysis and HCP screening using high resolution LC‐MS/MS revealed interesting results. Cell image analysis via TEM showed that lysosomes of GAA producer cell line appear full and engorged when compared to null cell line lysosomes. The HCP profile of the three cell lines capture step eluate was also compared using high resolution LC‐MS/MS to see whether HCP released during fermentation and harvest would show any significant difference across the three cell lines. As expected the majority of HCP are common to all three cell lines, a phenomenon widely reported in literature^9^, ^31^, ^32^; however, the GAA producer showed large abundance of four specific HCPs reported in the result section. At present, we are unable to determine whether this result is linked to GAA overexpression or alternatively could be due to a combination of mass spectrometer detector saturation which impaired quantitation of other species or also an effect of the competitive binding behavior caused by the presence of large amount of GAA in the feed of the GAA producer. The cathepsin B and Z proteases believed to be responsible for product degradation were found ubiquitously in all three cell lines suggesting that they do not co‐purifying with the products but are likely retained to some extent by the resin, or, that they co‐purify with another HCP that does interact with the resin. The data reported in this work provide a further understanding of the nature and relative concentrations of HCP impurities in biopharmaceutical samples and physical effects on the cell structure that target protein overexpression can have. These assays can be used as generic methods for HCP analysis in the biopharmaceutical industry.

## Conflict of Interest

The authors declare no conflicts of interest exist.

## Supporting information

Supporting Information 1
