# Supplementary material for: Effects of lysosomal biotherapeutic recombinant protein expression on cell stress and protease and general host cell protein release in Chinese hamster ovary cells
Source: Biotechnol Prog. 2017 Mar 29;33(3):666–76. doi: 10.1002/btpr.2455 (PMC5485175; doi:10.1002/btpr.2455)
Supplement: Supplementary file 1 — Supporting Information 1 [file BTPR-33-666-s001.docx]

| **Description** | **Accession ID** | **Confidence score** | **Anova (p)** | **pI** | **MW** | **GRAVY** | **Concentration (mg/L)** | | | **Percent total (%)** | | |
| --- | --- | --- | --- | --- | --- | --- | --- | --- | --- | --- | --- | --- |
|  |  |  |  |  |  |  | GAA | Null | mAb | GAA | Null | mAb |
| 14-3-3 protein theta | Q3SZI4 | 9.3 | 4.655E-08 | 4.68 | 27764.3 | -0.512 | 0.006 | 0.059 | 0.000 | 0.01 | 0.03 | 0.00 |
| 28 kDa heat- and acid-stable phosphoprotein | Q13442 | 17.2 | 4.482E-09 | 8.84 | 20630.0 | -1.612 | 0.005 | 0.111 | 0.003 | 0.00 | 0.05 | 0.02 |
| 28 kDa heat- and acid-stable phosphoprotein | Q3UHX2 | 17.7 | 6.999E-09 | 6.8 | 20604.9 | -1.606 | 0.000 | 0.013 | 0.000 | 0.00 | 0.01 | 0.00 |
| 30S ribosomal protein S6 | Q89MW3 | 9.8 | 4.197E-06 | 5.43 | 18627.6 | -0.987 | 0.141 | 0.162 | 0.020 | 0.15 | 0.07 | 0.11 |
| 40S ribosomal protein S6 | P47838 | 11.3 | 8.44E-08 | 10.83 | 28654.5 | -0.951 | 0.240 | 3.692 | 0.052 | 0.25 | 1.66 | 0.29 |
| 45 kDa calcium-binding protein | Q61112 | 44.5 | 1.04E-09 | 4.8 | 38308.5 | -0.765 | 0.422 | 3.854 | 0.073 | 0.44 | 1.73 | 0.42 |
| 4-hydroxy-tetrahydrodipicolinate synthase | A5D2Q5 | 7.8 | 1.259E-08 | 5.53 | 31588.3 | 0.084 | 0.002 | 0.128 | 0.001 | 0.00 | 0.06 | 0.00 |
| 78 kDa glucose-regulated protein | Q0VCX2 | 89.0 | 9.29E-08 | 5.01 | 70464.6 | -0.477 | 0.587 | 2.715 | 0.045 | 0.61 | 1.22 | 0.25 |
| Acetate kinase | Q6LLD4 | 4.8 | 0.0470184 | 5.72 | 42875.9 | 0.026 | 0.191 | 0.004 | 0.104 | 0.20 | 0.00 | 0.59 |
| Acidic phospholipase | P81243 | 14.8 | 0.0824282 | 4.58 | 14304.0 | -0.652 | 0.000 | 0.001 | 0.000 | 0.00 | 0.00 | 0.00 |
| Actin | P53689 | 25.0 | 1.591E-09 | 5.38 | 41622.6 | -0.177 | 7.386 | 0.099 | 0.000 | 7.66 | 0.04 | 0.00 |
| Actin, gamma | Q9UVW9 | 47.7 | 2.649E-07 | 5.45 | 41607.5 | -0.173 | 0.236 | 1.373 | 0.059 | 0.24 | 0.62 | 0.33 |
| Actin-10 | Q54GX7 | 44.4 | 7.769E-05 | 5.24 | 41746.7 | -0.204 | 0.273 | 0.353 | 0.005 | 0.28 | 0.16 | 0.03 |
| Actin-17 | Q554S6 | 20.8 | 2.88E-09 | 5.28 | 41571.4 | -0.209 | 0.311 | 1.475 | 0.056 | 0.32 | 0.66 | 0.31 |
| Actin-2 | Q9Y707 | 34.1 | 1.712E-08 | 5.31 | 41663.4 | -0.225 | 0.063 | 0.469 | 0.019 | 0.07 | 0.21 | 0.11 |
| Adapter molecule crk | P10365 | 42.4 | 5.051E-10 | 5.39 | 41635.4 | -0.206 | 0.034 | 0.674 | 0.000 | 0.04 | 0.30 | 0.00 |
| Adapter molecule crk | Q04929 | 15.4 | 1.305E-08 | 5.36 | 33805.6 | -0.658 | 0.005 | 0.074 | 0.000 | 0.01 | 0.03 | 0.00 |
| Angiopoietin-related protein 4 | Q9Z1P8 | 24.0 | 4.522E-09 | 8.21 | 43423.8 | -0.733 | 0.093 | 0.785 | 0.000 | 0.10 | 0.35 | 0.00 |
| Annexin A5 | P08758 | 44.9 | 1.806E-06 | 4.93 | 35805.6 | -0.33 | 0.181 | 0.314 | 0.027 | 0.19 | 0.14 | 0.15 |
| Astrocytic phosphoprotein PEA-15 | Q9Z297 | 6.2 | 1.073E-08 | 4.93 | 15040.1 | -0.644 | 0.003 | 0.132 | 0.000 | 0.00 | 0.06 | 0.00 |
| B box-binding protein | Q00436 | 11.4 | 0.0044479 | 9.35 | 34484.6 | -1.514 | 0.000 | 0.005 | 0.000 | 0.00 | 0.00 | 0.00 |
| Beta-1,4-glucuronyltransferase 1 | Q8BWP8 | 30.6 | 1.078E-07 | 6.84 | 47384.3 | -0.266 | 0.057 | 0.918 | 0.021 | 0.06 | 0.41 | 0.12 |
| Beta-1,4-glucuronyltransferase 1 | O43505 | 30.0 | 1.223E-07 | 6.77 | 47119.1 | -0.224 | 0.011 | 0.174 | 0.004 | 0.01 | 0.08 | 0.02 |
| Beta-actin-like protein 2 | Q562R1 | 11.4 | 3.103E-05 | 5.39 | 42003.2 | -0.19 | 0.022 | 0.004 | 0.000 | 0.02 | 0.00 | 0.00 |
| Bifunctional protease/dUTPase | P31625 | 5.0 | 9.515E-08 | 8.9 | 31311.1 | -0.094 | 0.001 | 0.098 | 0.000 | 0.00 | 0.04 | 0.00 |
| Bifunctional purine biosynthesis protein PurH | Q87VR9 | 21.6 | 1.286E-06 | 5.81 | 57463.4 | -0.049 | 0.008 | 0.359 | 0.001 | 0.01 | 0.16 | 0.01 |
| Bleomycin hydrolase | P70645 | 5.5 | 4.055E-09 | 6.04 | 52322.9 | -0.389 | 0.032 | 0.319 | 0.001 | 0.03 | 0.14 | 0.01 |
| Bone morphogenetic protein 1 | P13497 | 50.3 | 2.308E-07 | 6.26 | 98049.8 | -0.597 | 0.192 | 0.884 | 0.109 | 0.20 | 0.40 | 0.62 |
| cAMP-dependent protein kinase catalytic subunit gamma | P22612 | 5.5 | 5.278E-08 | 8.7 | 40303.3 | -0.339 | 0.475 | 4.676 | 0.016 | 0.49 | 2.10 | 0.09 |
| Cathepsin B | P10605 | 41.4 | 3.205E-08 | 5.23 | 27577.7 | -0.306 | 0.596 | 1.849 | 0.035 | 0.62 | 0.83 | 0.20 |
| Cathepsin Z | Q9R1T3 | 18.8 | 3.976E-07 | 5.51 | 27106.2 | -0.511 | 0.099 | 0.410 | 0.009 | 0.10 | 0.18 | 0.05 |
| Cell division control protein 42 homolog | Q2KJ93 | 15.2 | 4.434E-06 | 6.16 | 20933.2 | -0.222 | 0.001 | 0.107 | 0.000 | 0.00 | 0.05 | 0.00 |
| Chaperone protein DnaJ | Q9RUG2 | 10.4 | 0.0007799 | 5.62 | 40238.4 | -0.517 | 0.099 | 0.796 | 0.046 | 0.10 | 0.36 | 0.26 |
| Chaperone protein DnaK | P71331 | 9.8 | 1.309E-08 | 4.88 | 68262.9 | -0.432 | 0.062 | 0.272 | 0.043 | 0.06 | 0.12 | 0.25 |
| Chaperone protein DnaK | Q7UM31 | 19.2 | 1.103E-06 | 4.62 | 69422.7 | -0.414 | 0.047 | 0.843 | 0.017 | 0.05 | 0.38 | 0.09 |
| Chaperone protein dnaK2 | Q7U3C4 | 33.0 | 2.103E-07 | 4.71 | 67888.3 | -0.314 | 0.125 | 2.433 | 0.048 | 0.13 | 1.09 | 0.27 |
| Chemotaxis response regulator protein-glutamate methylesterase 1 | Q221I1 | 5.2 | 0.0003197 | 6.62 | 39828.4 | 0.182 | 0.033 | 0.675 | 0.011 | 0.03 | 0.30 | 0.06 |
| Chromosomal replication initiator protein DnaA | Q1I2G4 | 23.6 | 2.809E-09 | 6.73 | 57096.2 | -0.307 | 0.008 | 0.283 | 0.000 | 0.01 | 0.13 | 0.00 |
| Clusterin | P05371 | 96.5 | 2.741E-06 | 5.45 | 49076.4 | -0.534 | 3.451 | 7.611 | 0.180 | 3.58 | 3.42 | 1.02 |
| Clusterin | Q06890 | 94.4 | 7.644E-06 | 5.45 | 49344.6 | -0.554 | 0.707 | 1.736 | 0.032 | 0.73 | 0.78 | 0.18 |
| Clusterin (Fragment) | P14683 | 27.8 | 2.671E-05 | 5.68 | 22098.9 | -0.639 | 0.924 | 2.439 | 0.026 | 0.96 | 1.10 | 0.15 |
| Coiled-coil domain-containing protein 6 | Q16204 | 12.8 | 5.89E-10 | 6.95 | 53159.7 | -0.981 | 0.006 | 0.925 | 0.002 | 0.01 | 0.42 | 0.01 |
| Collagen alpha-1(VI) chain | Q04857 | 24.5 | 2.854E-06 | 5.15 | 106386.8 | -0.541 | 0.193 | 0.874 | 0.020 | 0.20 | 0.39 | 0.12 |
| Complement C3 | P01026 | 55.3 | 9.63E-08 | 6.12 | 184110.5 | -0.275 | 1.120 | 5.623 | 0.199 | 1.16 | 2.53 | 1.13 |
| Complement C3 | P01025 | 39.7 | 3.187E-07 | 6.06 | 184601.1 | -0.313 | 0.511 | 1.395 | 0.110 | 0.53 | 0.63 | 0.62 |
| Complement C3 | Q2UVX4 | 44.6 | 2.495E-11 | 6.37 | 185047.4 | -0.369 | 0.014 | 0.281 | 0.002 | 0.01 | 0.13 | 0.01 |
| Complement C3 | P12387 | 38.2 | 1.812E-07 | 6.47 | 184441.5 | -0.253 | 3.404 | 13.518 | 0.562 | 3.53 | 6.07 | 3.18 |
| Complement C3 | P01027 | 62.5 | 5.104E-07 | 6.3 | 184180.1 | -0.282 | 0.646 | 4.077 | 0.196 | 0.67 | 1.83 | 1.11 |
| Cytochrome b5 | P00171 | 13.7 | 9.103E-09 | 4.94 | 15197.9 | -0.516 | 0.015 | 0.470 | 0.001 | 0.02 | 0.21 | 0.01 |
| Cytochrome b6-f complex subunit 8 | Q06H04 | 5.8 | 1.692E-06 | 5.59 | 3197.9 | 1.628 | 0.000 | 0.000 | 0.000 | 0.00 | 0.00 | 0.00 |
| Cytochrome c2 | P86323 | 5.6 | 2.112E-09 | 9.3 | 11587.1 | -0.642 | 0.033 | 0.266 | 0.012 | 0.03 | 0.12 | 0.07 |
| D-alanine aminotransferase | Q5HF24 | 16.4 | 7.153E-05 | 4.95 | 31893.9 | -0.318 | 0.002 | 0.086 | 0.003 | 0.00 | 0.04 | 0.02 |
| Dermcidin | P81605 | 11.5 | 2.806E-05 | 5.64 | 9259.3 | -0.667 | 0.021 | 0.219 | 0.022 | 0.02 | 0.10 | 0.13 |
| Dihydropyrimidinase-related protein 2 | O02675 | 18.8 | 5.392E-05 | 5.95 | 62277.6 | -0.267 | 0.030 | 0.093 | 0.002 | 0.03 | 0.04 | 0.01 |
| Dipeptidyl peptidase 3 | Q99KK7 | 83.4 | 4.301E-06 | 5.26 | 82766.5 | -0.32 | 0.668 | 2.568 | 0.122 | 0.69 | 1.15 | 0.69 |
| Dolichyl-diphosphooligosaccharide--protein glycotransferas | P12244 | 15.7 | 1.604E-08 | 4.76 | 55052.9 | -0.505 | 0.019 | 0.138 | 0.001 | 0.02 | 0.06 | 0.00 |
| Doublesex- and mab-3-related transcription factor | Q3LH63 | 7.9 | 0.0021919 | 8.06 | 36999.2 | -0.765 | 0.017 | 0.000 | 0.000 | 0.02 | 0.00 | 0.00 |
| Drebrin | Q07266 | 9.3 | 7.445E-10 | 4.45 | 77340.8 | -0.782 | 2.896 | 0.814 | 0.002 | 3.00 | 0.37 | 0.01 |
| Dystroglycan | Q62165 | 107.6 | 5.09E-08 | 9.41 | 67629.1 | -0.376 | 1.021 | 6.132 | 0.181 | 1.06 | 2.75 | 1.03 |
| Dystroglycan | Q29243 | 66.1 | 5.891E-08 | 9.11 | 65832.4 | -0.345 | 0.035 | 0.296 | 0.010 | 0.04 | 0.13 | 0.05 |
| Dystroglycan | Q9TSZ6 | 65.2 | 4.8E-05 | 9.36 | 67762.3 | -0.404 | 0.005 | 0.074 | 0.002 | 0.00 | 0.03 | 0.01 |
| Dystroglycan | O18738 | 83.9 | 2.052E-07 | 9.33 | 67773.2 | -0.427 | 0.007 | 0.178 | 0.003 | 0.01 | 0.08 | 0.02 |
| Echinoderm microtubule-associated protein-like 2 | Q7TNG5 | 28.7 | 1.462E-08 | 5.83 | 70733.7 | -0.111 | 0.033 | 0.603 | 0.001 | 0.03 | 0.27 | 0.01 |
| EGF-containing fibulin-like extracellular matrix protein 1 | Q8BPB5 | 142.6 | 4.185E-10 | 4.96 | 53047.4 | -0.355 | 1.651 | 6.973 | 0.128 | 1.71 | 3.13 | 0.72 |
| EGF-containing fibulin-like extracellular matrix protein 1 | Q12805 | 128.4 | 1.629E-09 | 4.85 | 52765.1 | -0.346 | 0.363 | 0.038 | 0.025 | 0.38 | 0.02 | 0.14 |
| Elongation factor 1-alpha 1 | Q90835 | 22.7 | 1.251E-05 | 9.1 | 50156.9 | -0.267 | 0.041 | 0.165 | 0.046 | 0.04 | 0.07 | 0.26 |
| Elongation factor 2 | Q3SYU2 | 68.9 | 6.237E-05 | 6.42 | 95237.0 | -0.212 | 2.157 | 4.832 | 2.468 | 2.24 | 2.17 | 13.97 |
| Endoplasmic reticulum vesicle protein 25 | Q5B5L5 | 4.4 | 3.908E-07 | 6.14 | 21702.7 | -0.369 | 0.003 | 0.000 | 0.000 | 0.00 | 0.00 | 0.00 |
| Endoribonuclease YbeY | Q161G7 | 4.7 | 8.222E-09 | 4.26 | 17798.3 | 0.284 | 0.021 | 0.000 | 0.000 | 0.02 | 0.00 | 0.00 |
| Enolase (Fragment) | P42897 | 21.6 | 4.349E-10 | 5.43 | 42884.0 | -0.192 | 0.006 | 0.192 | 0.000 | 0.01 | 0.09 | 0.00 |
| Exosome complex component Rrp41 | A0RXU1 | 5.6 | 8.174E-05 | 5.04 | 26319.0 | -0.17 | 0.036 | 0.413 | 0.014 | 0.04 | 0.19 | 0.08 |
| F-actin-capping protein subunit alpha-1 | P47753 | 9.1 | 8.968E-08 | 5.34 | 32808.6 | -0.657 | 0.013 | 0.088 | 0.001 | 0.01 | 0.04 | 0.01 |
| F-box DNA helicase 1 | Q8NFZ0 | 27.9 | 0.0002112 | 8.58 | 117686.3 | -0.272 | 1.694 | 4.889 | 0.190 | 1.76 | 2.20 | 1.08 |
| Ferrochelatase | A9BEE9 | 14.6 | 7.699E-06 | 5.05 | 44606.0 | -0.186 | 0.615 | 0.038 | 0.457 | 0.64 | 0.02 | 2.59 |
| Follistatin-related protein 1 | Q58D84 | 74.9 | 3.903E-09 | 5.21 | 32593.5 | -0.609 | 0.807 | 2.899 | 0.202 | 0.84 | 1.30 | 1.14 |
| G kinase-anchoring protein 1 | Q5VSY0 | 5.1 | 8.707E-12 | 8.89 | 42078.1 | -1.201 | 0.000 | 0.600 | 0.000 | 0.00 | 0.27 | 0.00 |
| Gametocyte-specific factor 1-like | Q9CWD0 | 5.3 | 4.423E-05 | 8.12 | 17281.7 | -0.767 | 0.089 | 0.912 | 0.023 | 0.09 | 0.41 | 0.13 |
| Glia maturation factor beta | P60984 | 5.4 | 1.309E-07 | 5.19 | 16582.0 | -0.546 | 0.011 | 0.080 | 0.000 | 0.01 | 0.04 | 0.00 |
| Glyceraldehyde-3-phosphate dehydrogenase | P17244 | 54.7 | 7.054E-08 | 8.5 | 35616.7 | -0.084 | 0.071 | 0.335 | 0.001 | 0.07 | 0.15 | 0.01 |
| Glyceraldehyde-3-phosphate dehydrogenase 1 | O16027 | 12.1 | 1.467E-08 | 6.71 | 34579.6 | -0.055 | 0.046 | 0.381 | 0.023 | 0.05 | 0.17 | 0.13 |
| GTP-binding protein RHO1 | Q9HF54 | 16.0 | 1.502E-07 | 7.5 | 22734.2 | -0.271 | 0.004 | 0.028 | 0.001 | 0.00 | 0.01 | 0.00 |
| HD domain-containing protein 2 | Q0P565 | 10.1 | 2.828E-07 | 5.08 | 23192.2 | -0.573 | 0.004 | 0.110 | 0.000 | 0.00 | 0.05 | 0.00 |
| Heat shock 70 kDa | Q2TBX4 | 7.6 | 8.88E-09 | 5.23 | 49729.8 | -0.213 | 0.084 | 0.669 | 0.130 | 0.09 | 0.30 | 0.74 |
| Heat shock 70 kDa protein | Q05944 | 43.7 | 0.0008125 | 5.65 | 71467.8 | -0.406 | 0.134 | 0.923 | 0.003 | 0.14 | 0.41 | 0.02 |
| Heat shock 70 kDa protein | P16019 | 27.9 | 4.848E-08 | 5.31 | 71007.1 | -0.464 | 0.020 | 1.225 | 0.003 | 0.02 | 0.55 | 0.02 |
| Heat shock 70 kDa protein | P26791 | 27.8 | 9.798E-06 | 5.14 | 72051.8 | -0.394 | 0.001 | 0.041 | 0.027 | 0.00 | 0.02 | 0.16 |
| Heat shock 70 kDa protein | Q91233 | 45.8 | 0.0971841 | 5.41 | 70976.1 | -0.485 | 0.000 | 0.013 | 0.000 | 0.00 | 0.01 | 0.00 |
| Heat shock 70 kDa protein 13 | Q5R8D9 | 23.9 | 3.153E-06 | 5.52 | 49651.6 | -0.221 | 0.078 | 1.311 | 0.002 | 0.08 | 0.59 | 0.01 |
| Heat shock 70 kDa protein 1A | Q27975 | 30.3 | 8.365E-07 | 5.68 | 70127.3 | -0.402 | 0.049 | 0.107 | 0.000 | 0.05 | 0.05 | 0.00 |
| Heat shock 70 kDa protein 1A | Q61696 | 54.0 | 7.128E-07 | 5.52 | 69948.0 | -0.379 | 0.024 | 0.229 | 0.001 | 0.02 | 0.10 | 0.00 |
| Heat shock 70 kDa protein 4 | P11145 | 31.5 | 2.2E-10 | 5.24 | 71436.5 | -0.331 | 0.152 | 2.300 | 0.025 | 0.16 | 1.03 | 0.14 |
| Heat shock 70 kDa protein 6 | Q9N1U2 | 21.5 | 0.2963867 | 6.06 | 71156.3 | -0.449 | 0.007 | 0.084 | 0.014 | 0.01 | 0.04 | 0.08 |
| Heat shock 70 kDa protein cognate 1 | P29843 | 53.0 | 1.535E-07 | 5.33 | 70686.4 | -0.462 | 0.006 | 0.052 | 0.000 | 0.01 | 0.02 | 0.00 |
| Heat shock 70 kDa protein IV | Q06248 | 39.5 | 0.0003235 | 5.55 | 69749.8 | -0.428 | 0.040 | 0.562 | 0.070 | 0.04 | 0.25 | 0.40 |
| Heat shock cognate 70 kDa protein | Q24789 | 60.4 | 2.721E-09 | 5.66 | 72554.1 | -0.451 | 0.007 | 0.076 | 0.001 | 0.01 | 0.03 | 0.00 |
| Heat shock cognate 70 kDa protein 1 | P36415 | 43.2 | 1.576E-09 | 5.34 | 70414.6 | -0.489 | 0.032 | 0.140 | 0.002 | 0.03 | 0.06 | 0.01 |
| Heat shock cognate 70 kDa protein 3 | Q54BE0 | 54.5 | 1.163E-05 | 5.49 | 69947.0 | -0.461 | 0.654 | 1.857 | 0.018 | 0.68 | 0.83 | 0.10 |
| Heat shock cognate 71 kDa protein | P19378 | 215.5 | 9.552E-07 | 5.23 | 70673.7 | -0.448 | 0.146 | 3.922 | 0.021 | 0.15 | 1.76 | 0.12 |
| Heat shock cognate 71 kDa protein | P19120 | 229.4 | 1.941E-07 | 5.37 | 71109.3 | -0.454 | 0.076 | 0.782 | 0.011 | 0.08 | 0.35 | 0.06 |
| Heat shock cognate 71 kDa protein | O73885 | 165.6 | 8.95E-09 | 5.46 | 70827.0 | -0.449 | 0.015 | 0.398 | 0.001 | 0.02 | 0.18 | 0.01 |
| Heterogeneous nuclear ribonucleoprotein A/B | Q99020 | 23.7 | 7.245E-07 | 7.69 | 30831.3 | -0.893 | 0.001 | 0.297 | 0.000 | 0.00 | 0.13 | 0.00 |
| Heterogeneous nuclear ribonucleoprotein D0 | Q14103 | 5.8 | 1.427E-09 | 7.6 | 38303.0 | -0.932 | 0.007 | 0.309 | 0.000 | 0.01 | 0.14 | 0.00 |
| Heterogeneous nuclear ribonucleoprotein K | Q3T0D0 | 28.0 | 1.299E-08 | 5.14 | 51019.2 | -0.702 | 0.021 | 0.450 | 0.000 | 0.02 | 0.20 | 0.00 |
| Heterogeneous nuclear ribonucleoprotein Q | O60506 | 77.2 | 4.05E-08 | 8.68 | 69471.4 | -0.881 | 1.156 | 3.176 | 0.226 | 1.20 | 1.43 | 1.28 |
| Hsp90 co-chaperone Cdc37 | Q5EAC6 | 28.6 | 5.911E-07 | 5.09 | 44580.2 | -0.987 | 0.006 | 0.334 | 0.000 | 0.01 | 0.15 | 0.00 |
| Hsp90 co-chaperone Cdc37 | Q63692 | 39.5 | 7.95E-07 | 5.24 | 44510.4 | -0.969 | 0.002 | 0.195 | 0.001 | 0.00 | 0.09 | 0.00 |
| Ig gamma-4 chain C region | P01861 | 80.4 | 0.0001372 | 6.56 | 35940.6 | -0.423 | 0.001 | 0.032 | 3.104 | 0.00 | 0.01 | 17.58 |
| Ig kappa chain C region | P01834 | 32.9 | 1.686E-10 | 5.42 | 11608.9 | -0.553 | 0.001 | 0.000 | 0.823 | 0.00 | 0.00 | 4.66 |
| Importin-5 | O00410 | 94.0 | 1.47E-09 | 4.83 | 123498.7 | -0.134 | 0.754 | 4.259 | 0.070 | 0.78 | 1.91 | 0.39 |
| Insulin-like growth factor-binding protein 4 | P47879 | 35.4 | 8.08E-09 | 6.62 | 25760.3 | -0.535 | 0.037 | 0.919 | 0.017 | 0.04 | 0.41 | 0.10 |
| Isopentenyl-diphosphate delta-isomerase | A8AUV1 | 7.7 | 2.478E-10 | 5.39 | 37621.8 | -0.328 | 0.001 | 0.165 | 0.000 | 0.00 | 0.07 | 0.00 |
| Kin of IRRE-like protein 1 | Q96J84 | 15.6 | 1.146E-10 | 5.55 | 81714.6 | -0.404 | 0.011 | 0.139 | 0.000 | 0.01 | 0.06 | 0.00 |
| Lamin-B1 | P20700 | 13.6 | 2.487E-08 | 5.11 | 65961.7 | -0.811 | 0.009 | 0.180 | 0.000 | 0.01 | 0.08 | 0.00 |
| Legumain | O89017 | 31.6 | 3.451E-05 | 5.72 | 34818.2 | -0.368 | 0.433 | 1.425 | 0.318 | 0.45 | 0.64 | 1.80 |
| Lipoprotein lipase | Q06000 | 21.7 | 0.0027366 | 8.22 | 50298.0 | -0.422 | 0.068 | 0.282 | 0.002 | 0.07 | 0.13 | 0.01 |
| Lysine--tRNA ligase | P37879 | 51.6 | 3.691E-09 | 5.84 | 67859.0 | -0.418 | 0.038 | 0.906 | 0.001 | 0.04 | 0.41 | 0.01 |
| Lysosomal alpha-glucosidase | P10253 | 553.1 | 0.0155703 | 5.42 | 105323.7 | -0.129 | 34.791 | 5.319 | 0.491 | 36.07 | 2.39 | 2.78 |
| Lysosomal protective protein | P16675 | 50.6 | 9.753E-07 | 5.55 | 51395.2 | -0.321 | 0.800 | 5.823 | 0.223 | 0.83 | 2.62 | 1.26 |
| Lysyl oxidase homolog 1 | P97873 | 21.9 | 1.963E-08 | 6.17 | 56452.1 | -0.765 | 0.003 | 0.597 | 0.002 | 0.00 | 0.27 | 0.01 |
| Matrix metalloproteinase-19 | Q9JHI0 | 11.8 | 4.96E-08 | 8.87 | 46555.8 | -0.404 | 0.108 | 1.255 | 0.007 | 0.11 | 0.56 | 0.04 |
| Metalloproteinase inhibitor | P30120 | 5.3 | 1.384E-07 | 8.27 | 21536.8 | -0.109 | 0.051 | 0.185 | 0.011 | 0.05 | 0.08 | 0.06 |
| Methionine aminopeptidase 2 | P50579 | 10.5 | 7.263E-10 | 5.57 | 52760.4 | -0.687 | 0.001 | 0.161 | 0.000 | 0.00 | 0.07 | 0.00 |
| Multiple inositol polyphosphate phosphatase 1 | Q9Z2L6 | 5.6 | 2.332E-07 | 6.5 | 51546.9 | -0.389 | 0.006 | 0.365 | 0.000 | 0.01 | 0.16 | 0.00 |
| Myosin light chain 6B | Q8CI43 | 5.5 | 7.276E-09 | 5.41 | 22617.7 | -0.559 | 0.006 | 0.213 | 0.000 | 0.01 | 0.10 | 0.00 |
| Myotrophin | P62775 | 20.9 | 1.934E-07 | 5.28 | 12729.6 | -0.224 | 0.012 | 0.557 | 0.003 | 0.01 | 0.25 | 0.01 |
| Na(+)/H(+) exchange regulatory cofactor NHE-RF1 | P70441 | 22.7 | 8.103E-10 | 5.63 | 38468.9 | -0.75 | 0.011 | 0.247 | 0.000 | 0.01 | 0.11 | 0.00 |
| N-acetylglucosamine-6-sulfatase | Q8BFR4 | 15.2 | 1.816E-09 | 7.63 | 57632.5 | -0.411 | 0.075 | 0.208 | 0.001 | 0.08 | 0.09 | 0.01 |
| NAD-dependent protein deacylase | Q607X6 | 9.3 | 3.017E-09 | 5.98 | 27494.1 | -0.179 | 0.048 | 0.422 | 0.021 | 0.05 | 0.19 | 0.12 |
| Nascent polypeptide-associated complex subunit alpha, muscle-specific form | P70670 | 34.8 | 1.914E-09 | 9.39 | 220499.4 | -0.329 | 0.026 | 0.543 | 0.001 | 0.03 | 0.24 | 0.01 |
| Neudesin | Q9CQ45 | 19.3 | 1.953E-07 | 4.79 | 15612.4 | -0.674 | 0.008 | 0.222 | 0.000 | 0.01 | 0.10 | 0.00 |
| Neudesin | Q9UMX5 | 18.6 | 4.539E-08 | 4.81 | 15684.5 | -0.69 | 0.003 | 0.077 | 0.000 | 0.00 | 0.03 | 0.00 |
| Neudesin | Q6IUR5 | 18.5 | 8.449E-10 | 4.79 | 15684.5 | -0.62 | 0.002 | 0.071 | 0.000 | 0.00 | 0.03 | 0.00 |
| Neuraminidase | P67923 | 9.9 | 1.473E-07 | 6.82 | 51381.6 | -0.265 | 2.844 | 0.123 | 1.590 | 2.95 | 0.06 | 9.00 |
| Nidogen-1 | P10493 | 91.2 | 8.645E-08 | 5.24 | 133562.1 | -0.365 | 0.547 | 5.698 | 0.045 | 0.57 | 2.56 | 0.25 |
| Nidogen-1 | P14543 | 40.3 | 5.759E-09 | 5.05 | 133457.5 | -0.378 | 0.463 | 3.698 | 0.013 | 0.48 | 1.66 | 0.08 |
| Nuclear migration protein nudC | O35685 | 33.9 | 0.0016435 | 5.17 | 38358.0 | -1.061 | 0.007 | 0.073 | 0.000 | 0.01 | 0.03 | 0.00 |
| Nuclear migration protein nudC | Q63525 | 35.4 | 2.075E-05 | 5.27 | 38412.1 | -1.05 | 0.151 | 0.321 | 0.001 | 0.16 | 0.14 | 0.00 |
| Nuclear migration protein nudC | Q17QG2 | 24.2 | 6.283E-08 | 5.22 | 38242.9 | -1.028 | 0.024 | 0.426 | 0.000 | 0.02 | 0.19 | 0.00 |
| Nuclear transport factor 2 | Q32KP9 | 5.1 | 4.986E-07 | 5.1 | 14478.5 | -0.201 | 0.002 | 0.074 | 0.000 | 0.00 | 0.03 | 0.00 |
| Nuclease-sensitive element-binding protein 1 | P67808 | 40.7 | 1.79E-07 | 9.87 | 35793.0 | -1.495 | 0.033 | 0.727 | 0.029 | 0.03 | 0.33 | 0.16 |
| Nucleobindin-1 | Q63083 | 110.6 | 0.0021546 | 5.01 | 50919.5 | -1.022 | 0.727 | 1.260 | 0.036 | 0.75 | 0.57 | 0.20 |
| Nucleobindin-1 | Q02819 | 85.1 | 1.475E-09 | 4.96 | 50821.4 | -1.025 | 0.099 | 0.991 | 0.008 | 0.10 | 0.45 | 0.05 |
| Nucleobindin-1 | Q0P569 | 94.3 | 0.00041 | 5.05 | 52483.3 | -1.007 | 0.028 | 0.249 | 0.000 | 0.03 | 0.11 | 0.00 |
| Nucleobindin-2 | P81117 | 68.8 | 0.0001451 | 5.01 | 47423.9 | -1.085 | 0.615 | 1.571 | 0.036 | 0.64 | 0.71 | 0.21 |
| Nucleobindin-2 | Q9JI85 | 18.8 | 2.654E-07 | 4.96 | 47253.8 | -1.171 | 0.210 | 0.901 | 0.003 | 0.22 | 0.40 | 0.02 |
| Olfactomedin-like protein 3 | Q9NRN5 | 22.8 | 8.036E-11 | 6.18 | 43800.6 | -0.577 | 0.017 | 0.385 | 0.000 | 0.02 | 0.17 | 0.00 |
| Out at first protein homolog | Q8QZR4 | 5.2 | 4.213E-08 | 6.49 | 28462.4 | -0.257 | 0.073 | 0.836 | 0.000 | 0.08 | 0.38 | 0.00 |
| Peptide methionine sulfoxide reductase MsrA | A6WYH5 | 4.6 | 0.1352595 | 5.01 | 24158.1 | -0.416 | 0.073 | 0.027 | 0.027 | 0.08 | 0.01 | 0.15 |
| Peptidyl-prolyl cis-trans isomerase B | P80311 | 11.4 | 2.541E-07 | 9.13 | 20200.2 | -0.454 | 0.018 | 0.265 | 0.002 | 0.02 | 0.12 | 0.01 |
| Peroxiredoxin-1 | Q9JKY1 | 22.1 | 3.391E-10 | 8.21 | 22131.4 | -0.226 | 0.009 | 0.081 | 0.000 | 0.01 | 0.04 | 0.00 |
| Phosphoglycerate mutase 1 | P18669 | 90.6 | 7.735E-08 | 6.75 | 28672.7 | -0.498 | 0.094 | 3.083 | 0.027 | 0.10 | 1.38 | 0.15 |
| Phosphoglycerate mutase 1 | Q3SZ62 | 81.4 | 1.174E-07 | 6.44 | 28720.8 | -0.518 | 0.057 | 1.859 | 0.016 | 0.06 | 0.83 | 0.09 |
| Phosphoserine aminotransferase | B4SP45 | 4.4 | 0.0519085 | 5.74 | 38907.2 | 0.006 | 0.000 | 0.005 | 0.000 | 0.00 | 0.00 | 0.00 |
| Phosphoserine phosphatase | Q99LS3 | 17.0 | 6.447E-08 | 5.8 | 25095.9 | -0.116 | 0.003 | 0.155 | 0.000 | 0.00 | 0.07 | 0.00 |
| Pigment epithelium-derived factor | P97298 | 48.8 | 5.516E-08 | 6.45 | 44284.7 | -0.138 | 1.435 | 5.130 | 0.415 | 1.49 | 2.30 | 2.35 |
| Pigment epithelium-derived factor | P36955 | 35.6 | 9.811E-07 | 5.9 | 44387.8 | -0.164 | 1.297 | 0.966 | 0.184 | 1.34 | 0.43 | 1.04 |
| Plastin-2 | Q6P698 | 42.8 | 1.705E-09 | 5.26 | 69900.1 | -0.294 | 0.027 | 0.252 | 0.000 | 0.03 | 0.11 | 0.00 |
| Plastin-3 | O88818 | 72.7 | 7.127E-09 | 5.47 | 70795.0 | -0.331 | 0.070 | 1.525 | 0.001 | 0.07 | 0.68 | 0.01 |
| POTE ankyrin domain family member E | Q6S8J3 | 57.7 | 1.961E-05 | 5.83 | 121363.4 | -0.683 | 0.114 | 0.025 | 0.006 | 0.12 | 0.01 | 0.04 |
| Probable aquaporin PIP-type pTOM75 | Q6B411 | 5.0 | 8.2E-08 | 8.31 | 14962.2 | -0.647 | 0.023 | 0.071 | 0.009 | 0.02 | 0.03 | 0.05 |
| Probable dual-specificity RNA methyltransferase RlmN | A1A1I4 | 19.9 | 1.96E-08 | 6.84 | 42346.1 | -0.367 | 0.890 | 0.005 | 0.051 | 0.92 | 0.00 | 0.29 |
| Probable pectinesterase 30 | Q3EAY9 | 14.0 | 0.0008701 | 6.71 | 53060.4 | -0.24 | 0.007 | 0.269 | 0.000 | 0.01 | 0.12 | 0.00 |
| Procollagen C-endopeptidase enhancer 1 | Q61398 | 88.7 | 1.085E-07 | 8.63 | 47630.9 | -0.313 | 1.386 | 9.429 | 0.109 | 1.44 | 4.23 | 0.62 |
| Procollagen C-endopeptidase enhancer 1 | Q15113 | 25.2 | 3.573E-05 | 7.55 | 45549.5 | -0.286 | 0.620 | 0.575 | 0.129 | 0.64 | 0.26 | 0.73 |
| Procollagen-lysine,2-oxoglutarate 5-dioxygenase | Q63321 | 23.9 | 0.0005039 | 6.27 | 81646.8 | -0.4 | 0.045 | 0.477 | 0.020 | 0.05 | 0.21 | 0.11 |
| Procollagen-lysine,2-oxoglutarate 5-dioxygenase 1 | O77588 | 20.1 | 2.078E-08 | 6.17 | 81468.6 | -0.391 | 0.023 | 0.488 | 0.000 | 0.02 | 0.22 | 0.00 |
| Prolyl endopeptidase | Q9XTA2 | 53.4 | 6.122E-08 | 5.56 | 80641.4 | -0.353 | 0.096 | 0.332 | 0.002 | 0.10 | 0.15 | 0.01 |
| Protein crossbronx | B4HT57 | 13.4 | 0.0517061 | 5.29 | 28180.0 | -0.466 | 1.552 | 0.748 | 0.024 | 1.61 | 0.34 | 0.14 |
| Protein CYR61 | O00622 | 45.5 | 0.0017795 | 8.49 | 39438.4 | -0.381 | 0.975 | 1.586 | 0.715 | 1.01 | 0.71 | 4.05 |
| Protein disulfide-isomerase | Q8R4U2 | 74.8 | 1.078E-09 | 4.73 | 54997.9 | -0.453 | 0.104 | 0.982 | 0.008 | 0.11 | 0.44 | 0.05 |
| Protein disulfide-isomerase A3 | P38657 | 46.7 | 6.409E-08 | 5.78 | 54398.4 | -0.637 | 0.099 | 0.304 | 0.001 | 0.10 | 0.14 | 0.01 |
| Protein disulfide-isomerase A3 | P86235 | 124.9 | 2.691E-09 | 4.64 | 23387.9 | -0.55 | 0.070 | 0.754 | 0.010 | 0.07 | 0.34 | 0.06 |
| Protein disulfide-isomerase A3 | Q8JG64 | 67.9 | 1.585E-10 | 5.57 | 53782.7 | -0.557 | 0.021 | 0.357 | 0.013 | 0.02 | 0.16 | 0.07 |
| Protein S100-A13 | P79342 | 6.0 | 0.0145453 | 5.5 | 11198.9 | -0.434 | 0.002 | 0.029 | 0.000 | 0.00 | 0.01 | 0.00 |
| Proteinase K | P06873 | 12.9 | 1.012E-08 | 8.25 | 28906.8 | -0.218 | 0.014 | 0.187 | 0.001 | 0.01 | 0.08 | 0.00 |
| Purine nucleoside phosphorylase | P23492 | 23.1 | 1.864E-08 | 5.78 | 32277.1 | -0.132 | 0.009 | 0.645 | 0.001 | 0.01 | 0.29 | 0.01 |
| Putative gene 49 protein | O48403 | 5.1 | 0.0597136 | 4.79 | 13332.3 | -0.043 | 0.000 | 0.017 | 0.000 | 0.00 | 0.01 | 0.00 |
| Putative NADH dehydrogenase/NAD(P)H nitroreductase Reut_A1586 | Q471I1 | 9.1 | 3.522E-05 | 6.73 | 21531.5 | -0.239 | 0.056 | 0.003 | 0.001 | 0.06 | 0.00 | 0.00 |
| Putative phospholipase B-like 2 | Q4QQW8 | 15.8 | 1.061E-07 | 5.69 | 61943.4 | -0.265 | 0.244 | 1.197 | 0.041 | 0.25 | 0.54 | 0.23 |
| Pyruvate kinase PKM | P11980 | 32.8 | 7.584E-06 | 6.69 | 57686.6 | -0.096 | 0.048 | 0.714 | 0.022 | 0.05 | 0.32 | 0.12 |
| Pyruvate kinase PKM | P14618 | 38.4 | 3.493E-09 | 7.95 | 57805.7 | -0.132 | 0.046 | 0.379 | 0.007 | 0.05 | 0.17 | 0.04 |
| Ran-specific GTPase-activating protein | P34022 | 10.3 | 3.301E-10 | 5.15 | 23465.2 | -1.15 | 0.018 | 0.096 | 0.000 | 0.02 | 0.04 | 0.00 |
| Ras-like GTP-binding protein O-RHO | P22122 | 19.1 | 1.685E-07 | 5.82 | 21140.4 | -0.359 | 0.002 | 0.274 | 0.001 | 0.00 | 0.12 | 0.00 |
| Receptor-type tyrosine-protein phosphatase S | B0V2N1 | 52.0 | 8.29E-06 | 6.76 | 208981.4 | -0.39 | 0.163 | 1.467 | 0.002 | 0.17 | 0.66 | 0.01 |
| Recombination protein RecR | B3QYJ4 | 5.3 | 7.166E-07 | 5.61 | 22842.4 | -0.147 | 0.057 | 0.861 | 0.000 | 0.06 | 0.39 | 0.00 |
| Recombination protein RecR | Q2RZH8 | 4.8 | 1.929E-07 | 4.19 | 25489.1 | -0.38 | 0.003 | 0.227 | 0.001 | 0.00 | 0.10 | 0.00 |
| Retinoid-inducible serine carboxypeptidase | Q920A5 | 43.5 | 0.012753 | 5.25 | 47816.8 | -0.079 | 0.801 | 1.413 | 0.247 | 0.83 | 0.63 | 1.40 |
| Rho GDP-dissociation inhibitor 1 | Q5XI73 | 45.6 | 6.446E-07 | 5.1 | 23276.2 | -0.73 | 0.171 | 2.293 | 0.014 | 0.18 | 1.03 | 0.08 |
| Ribonuclease T2 | Q9CQ01 | 34.1 | 5.189E-09 | 5.92 | 26755.4 | -0.589 | 0.006 | 0.253 | 0.009 | 0.01 | 0.11 | 0.05 |
| Ribonucleoside-diphosphate reductase subunit M2 | Q60561 | 10.4 | 2.588E-10 | 5.25 | 44482.1 | -0.179 | 0.001 | 0.058 | 0.000 | 0.00 | 0.03 | 0.00 |
| Ribose-5-phosphate isomerase | P47968 | 41.3 | 6.096E-10 | 7.81 | 32450.9 | -0.233 | 0.001 | 0.137 | 0.001 | 0.00 | 0.06 | 0.00 |
| R-phycocyanin-1 beta chain | P37208 | 4.9 | 3.131E-08 | 4.92 | 18170.6 | 0.084 | 0.014 | 0.075 | 0.000 | 0.01 | 0.03 | 0.00 |
| Septin-2 | Q2NKY7 | 20.1 | 6.628E-09 | 6.15 | 41571.5 | -0.529 | 0.004 | 0.123 | 0.000 | 0.00 | 0.06 | 0.00 |
| Serine protease HTRA1 | Q92743 | 16.4 | 0.0001173 | 7.89 | 49048.1 | -0.202 | 0.020 | 0.051 | 0.000 | 0.02 | 0.02 | 0.00 |
| Serine/threonine-protein kinase PAK 1 | Q08E52 | 9.5 | 1.112E-10 | 5.63 | 60414.6 | -0.605 | 0.003 | 0.097 | 0.000 | 0.00 | 0.04 | 0.00 |
| Serum albumin | P02769 | 131.3 | n/a | 5.6 | 66433.0 | -0.429 | 0.276 | 0.358 | 0.387 | 0.29 | 0.16 | 2.19 |
| SH3 domain-binding glutamic acid-rich-like protein | O75368 | 10.7 | 7.369E-07 | 5.22 | 12774.3 | -0.691 | 0.002 | 0.027 | 0.001 | 0.00 | 0.01 | 0.00 |
| Small cysteine-rich outer membrane protein | B0B816 | 5.4 | 1.452E-08 | 5.13 | 7474.4 | -0.671 | 0.006 | 0.029 | 0.000 | 0.01 | 0.01 | 0.00 |
| Small ubiquitin-related modifier 2 | Q6LDZ8 | 7.4 | 1.241E-06 | 5.32 | 10608.9 | -0.893 | 0.126 | 1.137 | 0.041 | 0.13 | 0.51 | 0.23 |
| Soluble calcium-activated nucleotidase 1 | Q8VCF1 | 30.4 | 1.62E-08 | 6.28 | 45652.5 | -0.446 | 0.003 | 0.085 | 0.001 | 0.00 | 0.04 | 0.01 |
| Soluble calcium-activated nucleotidase 1 | Q8WVQ1 | 43.7 | 3.765E-08 | 5.72 | 44839.6 | -0.339 | 0.029 | 0.944 | 0.028 | 0.03 | 0.42 | 0.16 |
| Spermidine/putrescine import ATP-binding protein | A1TXH7 | 5.3 | 1.124E-07 | 4.92 | 42106.8 | -0.398 | 0.255 | 1.027 | 0.018 | 0.26 | 0.46 | 0.10 |
| Superoxide dismutase [Cu-Zn] 1 | O42724 | 4.6 | 0.00086 | 5.82 | 15757.2 | -0.444 | 0.000 | 0.008 | 0.000 | 0.00 | 0.00 | 0.00 |
| Suprabasin | A6QQF6 | 10.1 | 1.397E-07 | 6.74 | 54505.4 | -0.745 | 0.059 | 0.365 | 0.034 | 0.06 | 0.16 | 0.19 |
| Synaptic vesicle membrane protein VAT-1 | Q99536 | 5.8 | 2.518E-09 | 5.88 | 41789.1 | -0.043 | 0.051 | 0.231 | 0.000 | 0.05 | 0.10 | 0.00 |
| Taurocyamine kinase | P16641 | 17.5 | 6.933E-08 | 8.2 | 83891.0 | -0.366 | 0.017 | 0.178 | 0.146 | 0.02 | 0.08 | 0.83 |
| Tetranectin | P05452 | 28.5 | 2.312E-09 | 5.8 | 20138.9 | -0.484 | 0.034 | 0.426 | 0.000 | 0.04 | 0.19 | 0.00 |
| Thioredoxin | P10639 | 12.7 | 1.935E-08 | 4.8 | 11544.3 | -0.034 | 0.072 | 1.438 | 0.015 | 0.07 | 0.65 | 0.08 |
| Thioredoxin reductase 1, cytoplasmic | O62768 | 17.5 | 4.709E-06 | 6.07 | 54770.5 | -0.216 | 0.028 | 0.891 | 0.095 | 0.03 | 0.40 | 0.54 |
| Thioredoxin reductase 1, cytoplasmic | Q9JMH6 | 44.3 | 0.0003475 | 7.42 | 67083.8 | -0.233 | 0.042 | 1.087 | 0.138 | 0.04 | 0.49 | 0.78 |
| TIP41-like protein | O75663 | 14.6 | 1.011E-08 | 5.59 | 31444.0 | -0.401 | 0.001 | 0.040 | 0.000 | 0.00 | 0.02 | 0.00 |
| Tolloid-like protein 2 | Q9Y6L7 | 34.2 | 4.124E-11 | 5.8 | 97638.1 | -0.55 | 0.075 | 0.584 | 0.000 | 0.08 | 0.26 | 0.00 |
| Transcription initiation factor TFIID subunit 3 | Q9P6P0 | 19.0 | 1.134E-06 | 4.7 | 17681.0 | -0.32 | 0.073 | 0.394 | 0.004 | 0.08 | 0.18 | 0.02 |
| Transmembrane protein 132A | Q922P8 | 15.6 | 1.144E-07 | 5.37 | 106794.2 | -0.262 | 0.112 | 0.808 | 0.007 | 0.12 | 0.36 | 0.04 |
| Tripeptidyl-peptidase 1 | O89023 | 11.9 | 0.0003276 | 5.93 | 39877.6 | -0.173 | 0.082 | 0.550 | 0.016 | 0.09 | 0.25 | 0.09 |
| tRNA 2-thiocytidine biosynthesis protein TtcA | A3PNA9 | 15.3 | 0.0002483 | 6.26 | 33150.2 | -0.298 | 0.035 | 0.003 | 0.019 | 0.04 | 0.00 | 0.11 |
| Trypsin-1 | P16049 | 9.4 | 6.737E-08 | 6.36 | 23835.9 | -0.073 | 0.002 | 0.067 | 0.000 | 0.00 | 0.03 | 0.00 |
| Tryptophan--tRNA ligase, cytoplasmic | Q5R4J1 | 30.9 | 9E-10 | 5.99 | 53241.6 | -0.374 | 0.010 | 0.312 | 0.000 | 0.01 | 0.14 | 0.00 |
| Tryptophan--tRNA ligase, cytoplasmic | P32921 | 31.5 | 6.973E-09 | 6.44 | 54357.9 | -0.37 | 0.000 | 0.098 | 0.000 | 0.00 | 0.04 | 0.00 |
| Tubulin alpha-1 chain | O22347 | 6.0 | 5.552E-08 | 4.89 | 49731.1 | -0.191 | 0.002 | 0.183 | 0.001 | 0.00 | 0.08 | 0.00 |
| Twisted gastrulation protein homolog 1 | Q98T89 | 24.7 | 4.393E-06 | 4.97 | 22179.3 | -0.067 | 0.089 | 0.707 | 0.056 | 0.09 | 0.32 | 0.32 |
| U2 small nuclear ribonucleoprotein A' | P09661 | 20.7 | 2.356E-07 | 8.72 | 28284.4 | -0.496 | 0.310 | 1.203 | 0.103 | 0.32 | 0.54 | 0.58 |
| U2 small nuclear ribonucleoprotein A' | P57784 | 21.7 | 4.623E-07 | 8.72 | 28226.3 | -0.482 | 0.000 | 0.022 | 0.000 | 0.00 | 0.01 | 0.00 |
| Ubiquitin-conjugating enzyme E2 K | P61085 | 17.0 | 2.253E-09 | 5.33 | 22275.5 | -0.28 | 0.003 | 0.288 | 0.000 | 0.00 | 0.13 | 0.00 |
| Uncharacterized protein in bps2 5'region (Fragment) | P55031 | 27.3 | 0.2039077 | 4.92 | 51088.1 | -0.322 | 2.342 | 0.872 | 0.873 | 2.43 | 0.39 | 4.94 |
| UPF0134 protein MPN_139 | P75259 | 5.4 | 5.315E-09 | 9.15 | 19115.8 | -0.855 | 0.042 | 0.361 | 0.000 | 0.04 | 0.16 | 0.00 |
| UV excision repair protein RAD23 homolog B | P54727 | 18.2 | 5.843E-07 | 4.77 | 43171.2 | -0.388 | 0.010 | 0.185 | 0.002 | 0.01 | 0.08 | 0.01 |
| Vitamin K-dependent protein S | P53813 | 5.5 | 3.934E-11 | 4.99 | 69874.2 | -0.285 | 0.117 | 0.623 | 0.000 | 0.12 | 0.28 | 0.00 |
| V-type proton ATPase subunit S1 | Q9R1Q9 | 22.1 | 0.0003401 | 5.25 | 47737.6 | 0.149 | 0.050 | 0.429 | 0.043 | 0.05 | 0.19 | 0.24 |
| TOTAL (ng per 10μl sample) |  |  |  |  |  |  | 96.5 | 222.7 | 17.7 | 100 | 100 | 100 |

***Supporting data: HCP Full list comparison between GAA, Null and mAb CHO producer in IEX eluate.*** *Progenesis QIP software provided resulting data in the form of normalised abundance (molar equivalent) in triplicate readings of each species matched in the SwissProt database and the average of each triplicate was converted into concentration by dividing it by the specie molecular weight. Respective specie percentages were calculated based on relative amount of each specie out of the sum of amounts of all species in each 10 μl sample and were used as bubble graph data set (figure 6). Accession ID, confidence score and Anova (p) were provided by Progenesis QIP software. Isoelectric point was calculated using ExPASy pI calculator online tool (ExPASy. Compute pI/Mw tool). Hydropathicity index (GRAVY) was also calculated based on amino acidic sequence.*
